# Modulating Covalent Organic Framework Nanochannel Microenvironments for Sustainable Hydrogen Peroxide Photosynthesis

**DOI:** 10.1002/anie.7792190

**Published:** 2026-09-04

**Authors:** Ruixue Sun, Shaoyang Gao, Luqiao Feng, Peixuan Xie, Wenyu Shen, Nikita Rog, Marwan Mohammed, Qi Huang, Giulio Santori, Xianfeng Fan, Bien Tan, Cher Hon Lau

**Affiliations:** ^1^ School of Engineering University of Edinburgh Edinburgh UK; ^2^ Key Laboratory of Material Chemistry For Energy Conversion and Storage, Ministry of Education, Hubei Key Laboratory of Material Chemistry and Service Failure, School of Chemistry and Chemical Engineering Huazhong University of Science and Technology Wuhan P. R. China

**Keywords:** covalent organic frameworks (COFs), hydrogen peroxide (H_2_O_2_) photosynthesis, life cycle assessment (LCA), nanochannel microenvironment, side chain engineering

## Abstract

Photocatalytic hydrogen peroxide (H_2_O_2_) synthesis from water and oxygen is a promising alternative to the anthraquinone process, but its efficiency depends on charge separation and reactant transport. Herein, we report a side chain strategy to regulate nanochannel microenvironments of hydrazone‐linked covalent organic frameworks (COFs). Four COFs bearing H, OMe, OEt, or OEtOMe groups were prepared using the same benzotrithiophene node and hydrazone‐linked framework. The alkoxy side chains tune pore polarity, accessible porosity, electronic properties, and water and oxygen adsorption. Among them, COF‐Hz‐OEtOMe exhibits the strongest water and oxygen affinity and favorable charge‐separation behavior. It achieves an H_2_O_2_ production rate of 7240 µmol g^−1^ h^−1^ at the optimized catalyst loading in pure water and 22910 µmol g^−1^ h^−1^ with benzyl alcohol. A 1 L reactor using tap water and air accumulates 1.42 mM H_2_O_2_ after 4 h under natural sunlight. Experiments and theoretical calculations suggest that the conjugated framework promotes charge separation, while the alkoxy‐functionalized nanochannels improve reactant adsorption and oxygen activation. Life cycle assessment (LCA) highlights that natural sunlight and larger‐scale catalyst synthesis can reduce the calculated climate impact. These results demonstrate that pore‐wall side chain engineering provides an effective strategy for regulating charge behavior and reactant transport in porous photocatalysts.

## Graphical abstract

Alkoxy side chains regulate hydrazone‐linked COF nanochannel microenvironments by tuning pore polarity, reactant adsorption, and charge‐separation behavior. The optimized framework facilitates oxygen activation and hydrogen peroxide photosynthesis in real water and a 1 L natural‐sunlight reactor. LCA further shows that natural sunlight and larger‐scale catalyst synthesis reduce the calculated climate impact.

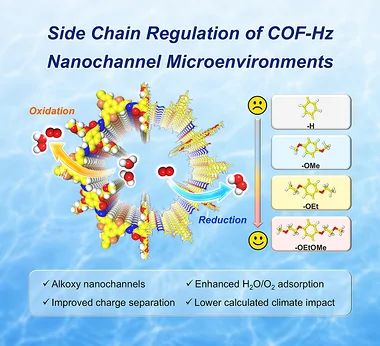

## 1 Introduction

Hydrogen peroxide (H_2_O_2_) is widely used in chemical synthesis, bleaching, and environmental remediation. Industrial H_2_O_2_ production mainly relies on the anthraquinone oxidation process, which requires high energy consumption, the use of noble metal catalysts, and the generation of hazardous organic waste, posing significant environmental challenges [1, 2]. Photocatalytic H_2_O_2_ production from water and oxygen under ambient conditions using solar energy is considered a sustainable and promising alternative for decentralized H_2_O_2_ synthesis [3, 4, 5]. However, efficient photocatalysis requires not only effective generation and separation of photogenerated charges, but also sufficient transport and activation of water and oxygen at the catalytic sites.

Covalent organic frameworks (COFs) are promising photocatalysts because of their crystalline structures, permanent porosity, and tunable electronic properties [6, 7]. To date, various strategies such as constructing donor‐acceptor (D‐A) systems [8], introducing heteroatoms [9], enhancing framework planarity [10], regulating linkage types [11], and building heterojunctions [12], have been developed to improve light harvesting and charge separation. In comparison, the effect of the nanochannel microenvironment on H_2_O_2_ production has received less attention. The pore‐wall structure affects the adsorption and diffusion of water and oxygen within COF channels. Functional groups on the pore walls can also change the local polarity and electronic structure of the framework. Therefore, changing a side chain may influence several properties at the same time, including crystallinity, porosity, reactant adsorption, and charge behavior. These combined effects should be considered when relating the molecular structure to photocatalytic activity [13, 14].

Post‐synthetic modification has been widely used to introduce functional side chains into COFs by pre‐installing reactive sites followed by grafting reactions [15, 16, 17]. Incomplete grafting and nonuniform functionalization often complicate the establishment of clear structure‐property relationships and may partially compromise the framework crystallinity required for efficient charge transport. A bottom‐up approach allows the side chains to be introduced during COF synthesis. Previous studies have demonstrated that side‐chain engineering can regulate the pore microenvironment of COFs for photocatalytic H_2_O_2_ production. For example, Xie et al. showed that pore‐wall side chains can modulate hydrophilicity and charge‐transfer behavior in hydrazone‐linked COFs [17]. Building on these findings, a more systematic understanding of how side‐chain structure simultaneously affects charge dynamics, accessible porosity, reactant adsorption, and oxygen activation remains important.

In this work, we report a rational bottom‐up strategy to engineer the pore‐wall microenvironment of hydrazone‐linked COFs for photocatalytic H_2_O_2_ production. By coupling an electron‐rich benzotrithiophene (BTT) node with dihydrazide linkers bearing systematically varied substituents (H, OMe, OEt, and OEtOMe), we constructed a series of isoreticular COF‐Hzs with tunable pore surfaces. This well‐defined model system enables the role of pore‐wall side chains to be examined without changing the overall framework topology. The resulting COFs exhibit distinct crystallinity, accessible porosity, nanochannel polarity, water and oxygen affinity, and electronic properties. Among them, COF‐Hz‐OEtOMe delivers the highest H_2_O_2_ production activity despite its lower BET surface area than COF‐Hz‐OMe, indicating that nanochannel functionality rather than surface area governs photocatalytic performance. Experimental studies and theoretical calculations reveal a division of function in which the conjugated framework governs charge dynamics, whereas the alkoxy‐functionalized nanochannels enrich water and oxygen and facilitate oxygen activation. The optimized material further maintains high activity in real water, under air, and in a 1 L reactor under natural sunlight. Life cycle assessment (LCA) was employed to systematically evaluate the environmental impact of H_2_O_2_ production under different irradiation conditions, further confirming the sustainability advantages of the solar‐driven photocatalytic system.

## 2 Results and Discussion

### 2.1 Design and Structure Characterization of COF‐Hzs

As illustrated in Figure 1a, four hydrazone‐linked COFs were synthesized by the solvothermal condensation of benzo[1,2‐b:3,4‐b′:5,6‐b′′]trithiophene‐2,5,8‐tricarbaldehyde with terephthalohydrazide or its derivatives (2,5‐dimethoxyterephthalohydrazide, or 2,5‐diethoxyterephthalohydrazide, or 2,5‐bis(2‐methoxyethoxy)terephthalohydrazide). The hydrazone linkage provides a conjugated framework, while the side chains are located within the 1D channels. This series was used to examine the combined effects of pore‐wall substitution on framework properties, reactant adsorption, and photocatalytic performance.

**FIGURE 1:**
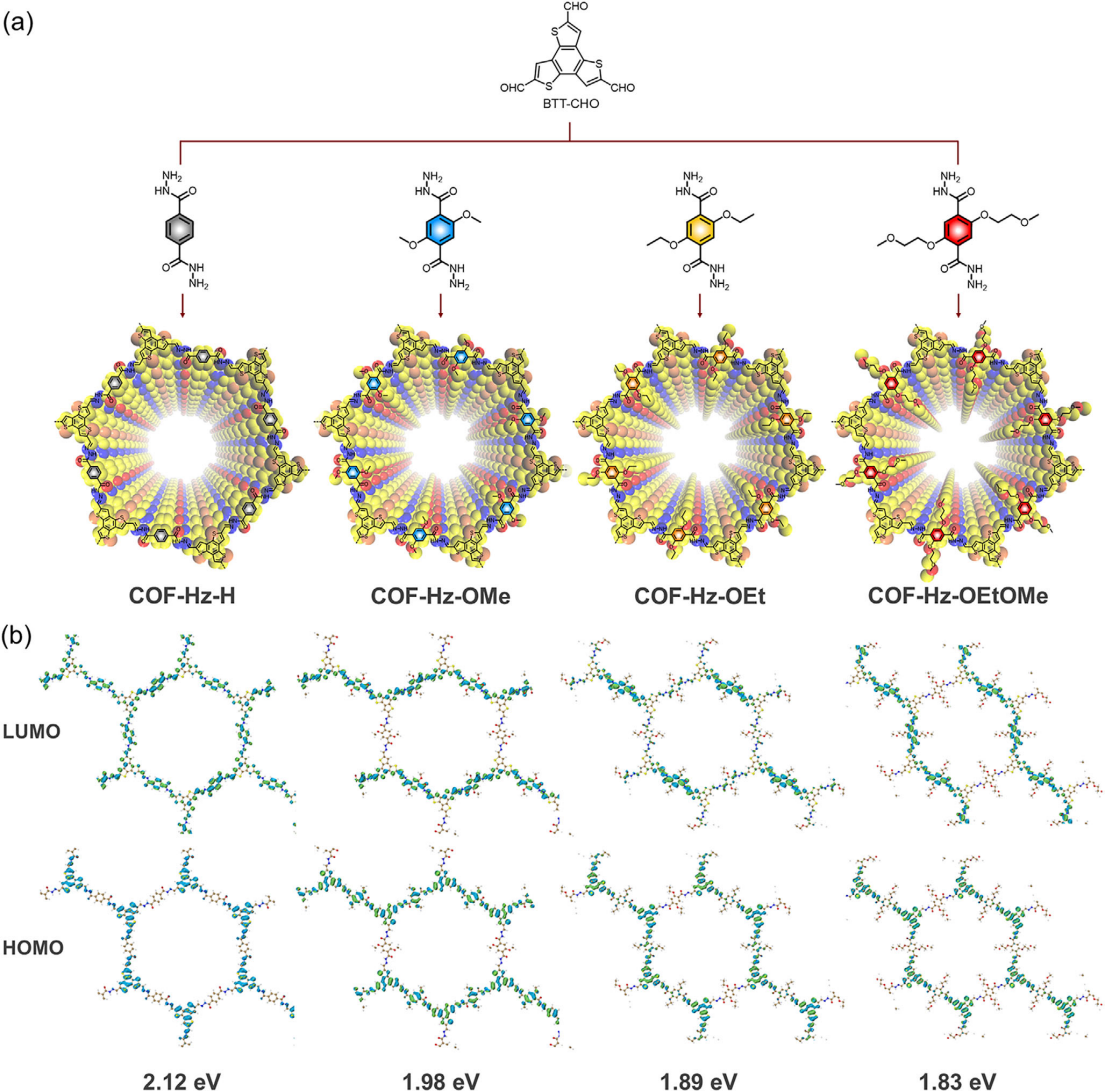
(a) Bottom‐up synthesis of the COF‐Hz series with systematically varied pore‐wall substituents. (b) Calculated frontier‐orbital distributions for simplified COF‐Hz models.

Prior to experimental synthesis, density functional theory (DFT) calculations were employed to evaluate the electronic structures of the target COF‐Hzs (Figure 1b). In all four models, the highest occupied molecular orbitals (HOMOs) are primarily localized on the electron‐rich thiophene‐based moieties, whereas the lowest unoccupied molecular orbitals (LUMOs) are distributed over the linker‐derived phenyl regions. The frontier‐orbital distribution of COF‐Hz‐OEtOMe also suggests reduced electron‐hole overlap. This spatial separation suggests that visible‐light excitation can induce an intramolecular charge transfer from the thiophene units (HOMO) to the benzene‐based acceptors (LUMO) via the hydrazone linkages. Notably, the calculated HOMO–LUMO gap decreases as the alkoxy substituent is extended, from 2.12 eV for COF‐Hz‐H to 1.83 eV for COF‐Hz‐OEtOMe. Because static orbital plots alone cannot establish charge‐separation or recombination kinetics, these predictions are evaluated below by temperature‐dependent photoluminescence, transient photocurrent, impedance, and lifetime measurements [18].

The crystalline nature and long‐range periodicity of the synthesized COFs were confirmed via powder x‐ray diffraction (PXRD) analysis. No reflections from the monomers were detected (Figure S1). The long‐range periodicity of the COF‐Hzs was successfully indexed to the *P3* space group, confirming a hexagonal lattice arrangement. The refined unit cell parameters are summarized as follows: for COF‐Hz‐H, *a* = *b* = 37.1367 Å, *c* = 3.5415 Å, and *α* = *β* = 90°, *γ* = 120°; for COF‐Hz‐OMe, *a* = *b* = 37.2063 Å, *c* = 3.5256 Å, and *α* = *β* = 90°, *γ* = 120°; for COF‐Hz‐OEt, *a* = *b* = 37.2020 Å, *c* = 3.5411 Å, and *α* = *β* = 90°, *γ* = 120°; for COF‐Hz‐OEtOMe, *a* = *b* = 37.1984 Å, *c* = 3.5466 Å, and *α* = *β* = 90°, *γ* = 120° (detailed in Table S1). Notably, the experimental PXRD patterns exhibit a prominent diffraction peak at 2.7° 2*θ*, assigned to the (100) plane, alongside a broad signal at approximately 25° 2*θ* corresponding to the (001) plane. This broad reflection is characteristic of π‐π stacking interactions between the COF layers, indicating a degree of long‐range order with a specific interlayer distance along the *c*‐direction. Similarly, COF‐Hz derivatives with different alkoxy side chains display consistent diffraction profiles, with peaks appearing at identical positions corresponding to the same crystallographic planes. This observation suggests that the side chain functionalization does not alter the underlying framework symmetry. The experimental results showed excellent agreement with the simulated PXRD patterns based on the eclipsed AA stacking mode, as illustrated in Figures 2a–d, S2 and S3. High‐resolution transmission electron microscopy (HRTEM) imaging further revealed clear lattice fringes with a spacing of approximately 0.35 nm for the alkoxy‐functionalized COF‐Hz‐OMe, COF‐Hz‐OEt, and COF‐Hz‐OEtOMe (Figure S4), corresponding to the (001) plane associated with interlayer π–π stacking and consistent with the PXRD results.

**FIGURE 2:**
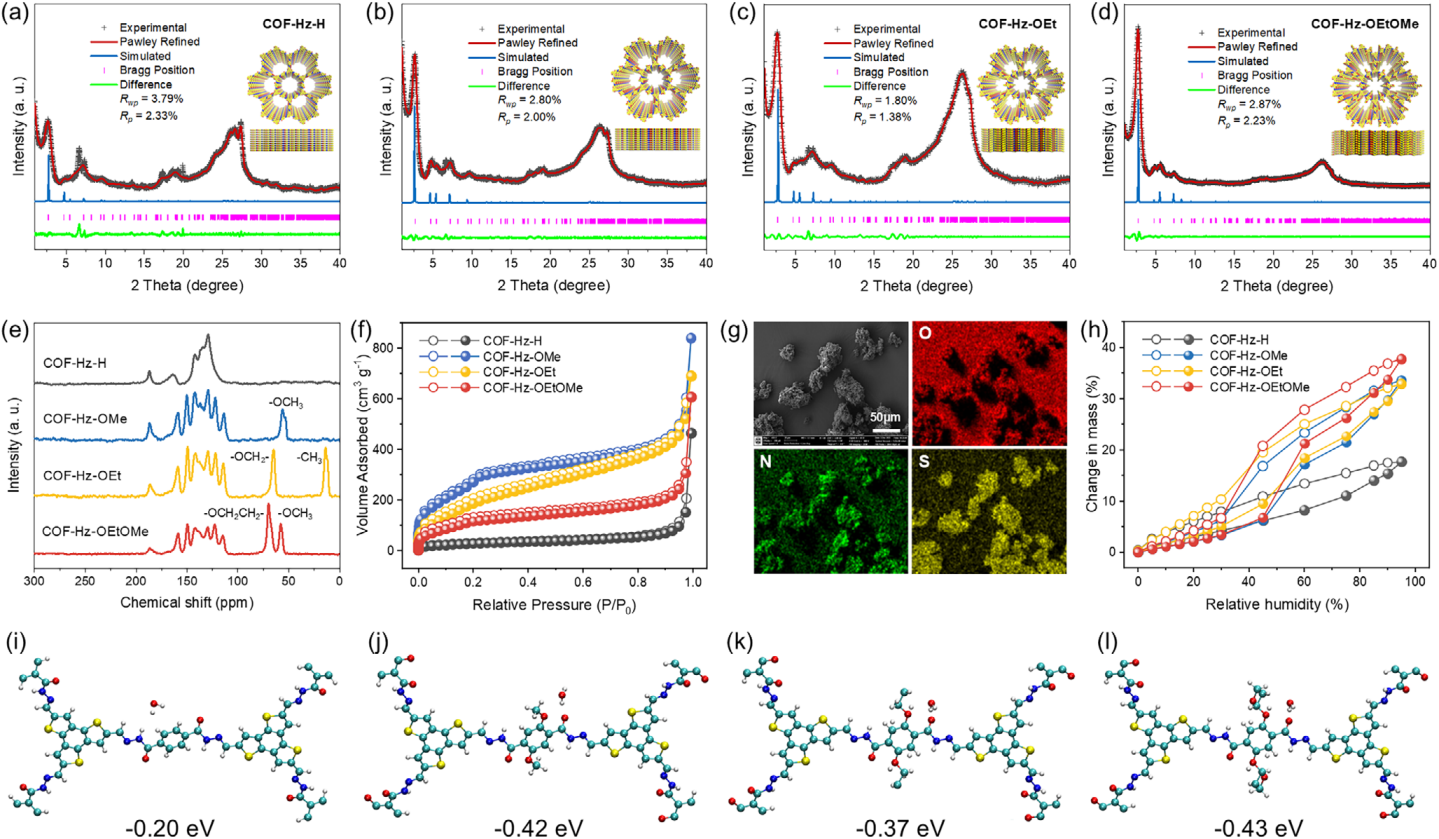
Structural and pore‐environment characterization of COF‐Hz‐H, COF‐Hz‐OMe, COF‐Hz‐OEt, and COF‐Hz‐OEtOMe: (a‐d) PXRD refinements, (e) Solid‐state NMR spectra and (f) N_2_ sorption isotherms of COF‐Hzs. (g) SEM image and EDS mapping of COF‐Hz‐OEtOMe. (h) The water vapor adsorption isotherms of COF‐Hzs. (i–l) DFT calculated water vapor adsorption sites and corresponding binding energies.

The chemical structures of the hydrazone‐linked COFs were systematically characterized via Fourier transform infrared (FT‐IR) and solid‐state ^13^C cross‐polarization magic angle spinning (^13^C CP/MAS) NMR spectroscopy. As illustrated in Figure S5, the FT‐IR spectra exhibit a distinctive absorption band at approximately 1600 cm^−1^, which is assigned to the C═N stretching vibration [19]. The emergence of this peak, coupled with the disappearance of the characteristic –CHO stretching bands, confirms the successful Schiff‐base condensation between the hydrazide and aldehyde precursors. Furthermore, the retention of N─H and amide C═O stretching vibrations at ∼3275 cm^−1^ and ∼1660 cm^−1^, respectively, demonstrates that the core hydrazone linkages remain intact within the resulting COF‐Hz frameworks. Successful condensation was further verified by ^13^C CP/MAS NMR spectroscopy (Figure 2e). The characteristic C═N resonance appeared at 142 ppm, accompanied by the amide carbonyl signal at 164 ppm. The absence of aldehyde resonances in the 190∼200 ppm range indicates the completion of the Schiff‐base reaction. For functionalized COFs, the alkoxy carbon signals were clearly identified between 0 ppm and 80 ppm [20].

X‐ray photoelectron spectroscopy (XPS) was further employed to examine the surface chemical composition and chemical environments of the COF‐Hzs (Figure S6). The high‐resolution C 1s spectra exhibit components at approximately 284.8, 286.1∼286.3, 287.3∼287.7, and 289.8∼290.1 eV, assigned to C─C/C═C, C═N/C─S/C–O, O═C–N, and π–π* interactions, respectively. The N 1s spectra are dominated by the C═N and ─NH─C═O environments at 399.8∼400.1 eV, together with a weaker hydrogen‐bonded N contribution at 401.5∼402.3 eV. In the O 1s spectra, the C═O component appears at 531.0∼531.3 eV, while the component at 532.5∼533.2 eV is associated with C─O─C and/or hydrogen‐bonded carbonyl environments. A weak higher‐binding‐energy contribution at 534.5∼535.5 eV is assigned to adsorbed H_2_O in the alkoxy‐functionalized COFs. The S 2p spectra show the characteristic thiophene‐S 2p_3/2_ and S 2p_1/2_ doublet at 163.8∼164.0 and 165.0∼165.2 eV, respectively, together with a minor high‐binding‐energy S contribution. These XPS results are consistent with the proposed framework structures and further support the successful incorporation of the pore‐wall side chains.

Elemental analysis (EA) of the COF‐Hz series shows that the observed carbon, hydrogen, and nitrogen contents are generally consistent with the theoretical values (Table S2). A slight excess of oxygen was detected, which can be primarily attributed to the adsorption of atmospheric moisture and oxygen within the highly porous framework during the sample handling and weighing process. Given the large surface area of these COFs, such minor deviations in oxygen content are frequently observed in similar porous crystalline materials and do not compromise the structural integrity verified by FT‐IR, NMR and XPS results [21, 22].

Chemical stability of the COF‐Hzs was further evaluated by immersing the samples in DMF, THF, methanol, and acetone for three days. COF‐Hz‐OMe, COF‐Hz‐OEt, and COF‐Hz‐OEtOMe largely retained their characteristic PXRD patterns, with only a slight decrease in the intensity of the (100) reflection, whereas COF‐Hz‐H showed a more pronounced loss of long‐range order owing to its relatively low initial crystallinity (Figure S7). In all cases, the characteristic FT‐IR bands remained essentially unchanged after solvent treatment, confirming the preservation of the hydrazone‐linked chemical structures (Figure S8).

The porosity of the COF‐Hz series was evaluated via N_2_ adsorption‐desorption isotherms at 77 K (Figure 2f). COF‐Hz‐H exhibited a much lower Brunauer–Emmett–Teller (BET) specific surface area (100 m^2^ g^−1^) than the derivatives containing alkoxy groups, which can be attributed to its high flexibility and free rotation that leads to poor molecular packing or partial pore collapse. In contrast, the introduction of a methoxy group (COF‐Hz‐OMe) triggered a dramatic 10‐fold increase in surface area (1097 m^2^ g^−1^), demonstrating that the alkoxy side chains effectively increase framework rigidity and help maintain the pore structure. These observations are consistent with previous reports on similar materials [11, 23]. However, as the side chain length further increased, the surface area decreased by 30% for COF‐Hz‐OEt (770 m^2^ g^−1^) and 59% for COF‐Hz‐OEtOMe (451 m^2^ g^−1^). This reduction is primarily due to the longer alkoxy chains occupying more significant volume within the nanochannels despite their role in stabilizing the framework.

Pore size distributions were calculated using nonlocal density functional theory (NLDFT), as shown in Figure S9. The dominant pore sizes were 1.4 nm for COF‐Hz‐H, 1.4 to 2.4 nm for COF‐Hz‐OMe, 1.4 to 2.0 nm for COF‐Hz‐OEt, and 1.4 to 1.8 nm for COF‐Hz‐OEtOMe. These results show an overall decrease in the accessible pore width with increasing alkoxy side‐chain length, consistent with the greater occupation of the nanochannels by the longer side chains and with previous reports [24, 25].

Scanning electron microscopy (SEM) images reveal that the COF‐Hzs consist of irregularly‐sized nanoparticles (Figures 2g and S10–S12). EDS mapping showed the uniform distribution of nitrogen, sulfur, and oxygen within the COF particles, while the oxygen signal observed in the background originated from the oxygen‐containing carbon adhesive tabs used as the substrate. Furthermore, thermogravimetric analysis (TGA) was conducted to evaluate the thermal stability of the materials. The results show that the COF‐Hzs remain stable up to 350°C (Figure S13), indicating their robust structural integrity under thermal stress.

The hydrophilicity of the COF‐Hzs was evaluated by the measurement of water vapor adsorption performance at 25°C, as shown in Figure 2h. At the relative humidity (RH) of 95%, the water vapor uptake ability was 17.68% (9.81 mmol g^−1^) for COF‐Hz‐H, 33.53% (18.61 mmol g^−1^) for COF‐Hz‐OMe, 32.84% (18.23 mmol g^−1^) for COF‐Hz‐OEt, 37.70% (20.93 mmol g^−1^) for COF‐Hz‐OEtOMe. Notably, compared to the pristine COF‐Hz‐H, which shows negligible water uptake across the investigated humidity range, the alkoxy‐functionalized COF‐Hzs exhibit a sharp increase beyond 45% RH. This disparity signifies a transition from surface‐bound adsorption to rapid pore‐filling, characteristic of improved surface affinity. To bridge the gap between macroscopic adsorption behavior and molecular‐level interactions, we performed DFT calculations to quantify the binding strength of water molecules onto the COF frameworks. The calculated minimum adsorption energies for COF‐Hz‐H, COF‐Hz‐OMe, COF‐Hz‐OEt, and COF‐Hz‐OEtOMe were −0.20, −0.42, −0.37, and −0.43 eV, respectively, with the adsorption sites localized in the vicinity of the benzene substituents (Figure 2i–l). The more negative adsorption energies observed for the alkoxy‐functionalized COF‐Hzs indicate a stronger affinity between the framework and water molecules. This enhancement is attributed to the alkoxy side chains, which act as efficient hydrogen‐bond acceptors [24, 26]. Consequently, the incorporation of these hydrophilic groups significantly improves the water adsorption capacity, thereby strengthening the interaction between the photocatalyst surface and the aqueous reaction medium.

### 2.2 Charge Separation and Electronic Structure Modulation

The influence of pore‐wall substitution on charge generation and separation was examined using complementary optical and electrochemical measurements. Temperature‐dependent photoluminescence spectroscopy was first used to estimate the exciton binding energy (*E_b_*), for which a lower value indicates more facile exciton dissociation (Figures 3a and S14). The *E_b_* values were calculated by fitting the experimental data to the Arrhenius equation: *I(T)* = *I_0_/[1 + Aexp(‐E_b_/k_B_T)]* [27]. Fitting the temperature‐dependent intensities gives *E_b_* values of 122.49, 73.73, 66.18, and 48.56 meV for COF‐Hz‐H, COF‐Hz‐OMe, COF‐Hz‐OEt, and COF‐Hz‐OEtOMe, respectively. Thus, all three alkoxy‐functionalized frameworks show substantially lower apparent binding energies than the unsubstituted COF. This reduction indicates that the introduction of side chains effectively lowers the energy barrier for exciton separation and enhances the efficiency of internal exciton dissociation. Consistent with this trend, the substituted materials exhibit higher transient photocurrent responses and smaller fitted impedance arcs than COF‐Hz‐H (Figure 3b,c). Time‐resolved photoluminescence measurements further show longer average lifetimes for the alkoxy‐functionalized COF‐Hzs (Figures 3d and S15). Although each technique probes a different aspect of the excited‐state and interfacial response, their common trend is consistent with alkoxy substitution facilitating exciton dissociation, charge transport, and longer‐lived charge‐separated states. These changes increase the population of charges available for surface reactions and provide a photophysical basis for the catalytic trends discussed below.

**FIGURE 3:**
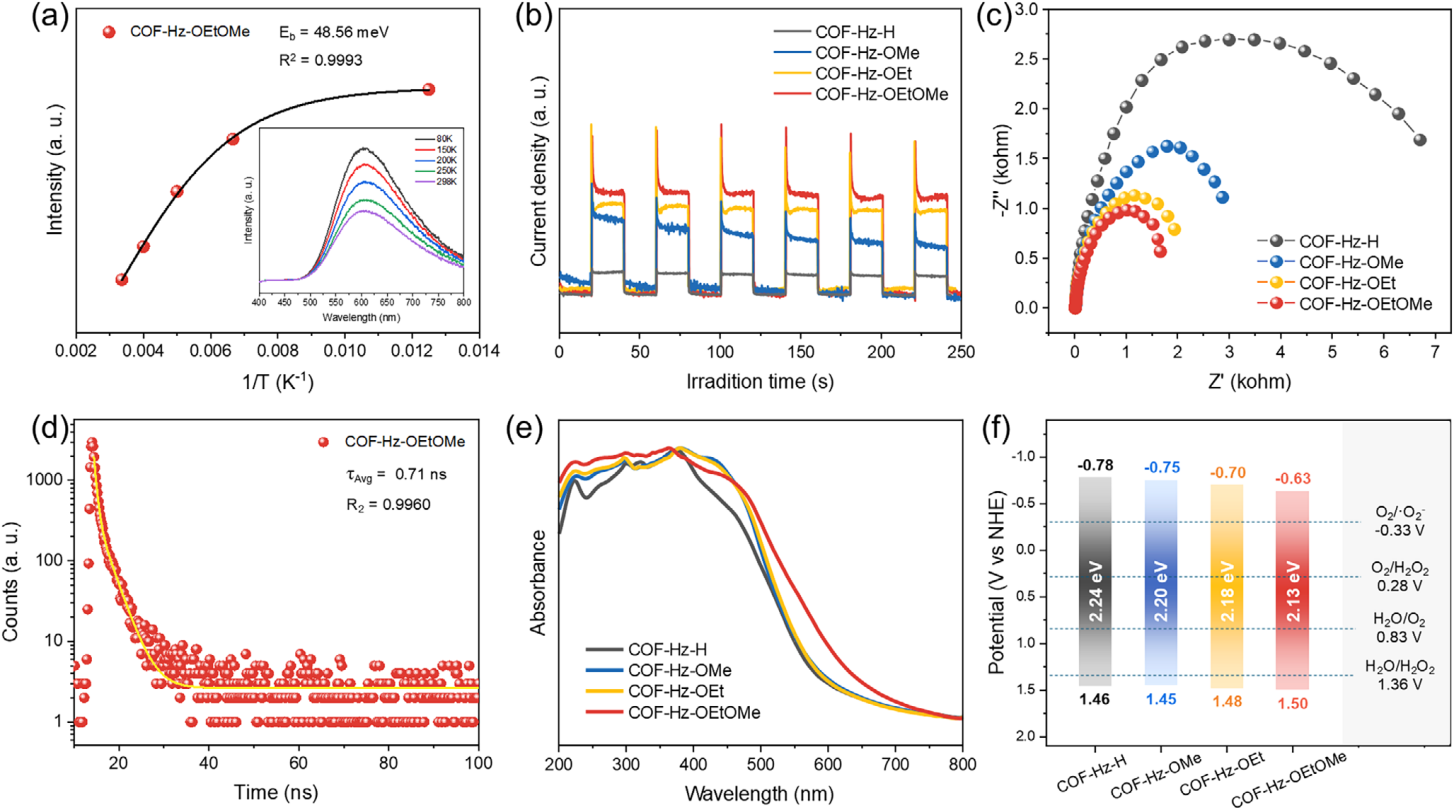
(a) Temperature‐dependent photoluminescence spectra of COF‐Hz‐OEtOMe. (b) Transient photocurrent responses under visible light irradiation and (c) fitting Nyquist plot using equivalent circuits from electrochemical impedance spectroscopy (EIS) of COF‐Hzs. (d) Time‐dependent photoluminescence (PL) decay spectra of COF‐Hz‐OEtOMe. (e) Solid‐state UV‐Vis absorption spectra of COF‐Hzs. (f) Energy diagram of COF‐Hzs.

Ultraviolet‐visible (UV‐Vis) diffuse‐reflectance spectra revealed that COF‐Hzs exhibit broad visible‐light absorption capabilities. As shown in Figure 3e, the absorption edges were determined to be 705 nm, 718 nm, 724 nm, and 741 nm for COF‐Hz‐H, COF‐Hz‐OMe, COF‐Hz‐OEt, and COF‐Hz‐OEtOMe, respectively. Accordingly, the optical band gaps (*E_g_*) calculated via Tauc plots (Figure S16) are 2.24, 2.20, 2.18, and 2.13 eV, respectively. Mott‐Schottky analysis gives flat‐band potentials of −0.78, −0.75, −0.70, and −0.63 V vs normal hydrogen electrode (NHE) for COF‐Hz‐H, COF‐Hz‐OMe, COF‐Hz‐OEt, and COF‐Hz‐OEtOMe, respectively (Figure S17). As the flat‐band potential of an n‐type semiconductor is generally close to its conduction‐band edge, these values were used to approximate the LUMO potentials. Combined with the optical band gaps, the corresponding highest occupied molecular orbital (HOMO) potentials were calculated to be +1.46, +1.45, +1.48, and +1.50 V vs NHE, respectively. As illustrated in Figure 3f, the band positions of the COF‐Hzs are thermodynamically favorable for H_2_O_2_ photosynthesis. Specifically, the LUMO potentials are sufficiently negative to drive the oxygen reduction reaction (ORR, E(O_2_/·O_2_^−^) = −0.33 V and E(O_2_/H_2_O_2_) = +0.28 V vs NHE at pH 7), while the HOMO potentials are sufficiently positive to drive the water oxidation reaction (WOR, E(H_2_O/H_2_O_2_) = +1.36 V and E(H_2_O/O_2_) = +0.83 V vs NHE at pH = 7).

### 2.3 Photocatalytic H_2_O_2_ Production

The photocatalytic H_2_O_2_ production performance of the COF‐Hzs was evaluated in pure water saturated with O_2_ under visible light irradiation (PLS‐SXE300E, High Power Xenon Light Source, Beijing Perfectlight Technology Co., Ltd.). The content of the generated H_2_O_2_ was determined via iodometric titration, monitoring the absorbance at 351 nm using a UV‐vis spectrophotometer. Control experiments conducted in the dark for the initial 20 min showed no detectable generation of H_2_O_2_ (Figure 4a). Upon visible light irradiation (λ ≥ 420 nm), the amount of accumulated H_2_O_2_ increased linearly over a period of 2 h. During the first hour, the average H_2_O_2_ production rates for the alkoxy‐functionalized COF‐Hz‐OMe, COF‐Hz‐OEt, and COF‐Hz‐OEtOMe were determined to be 4450, 4850, and 5230 µmol g^−1^ h^−1^, respectively. These values significantly surpass the photocatalytic performance of the pristine COF‐Hz‐H (1520 µmol g^−1^ h^−1^). Notably, as shown in Figure S18, no H_2_O_2_ generation was observed when the constituent monomers were employed as photocatalysts under identical conditions. This result demonstrates the crucial role of the COF framework structure in photocatalytic H_2_O_2_ production.

**FIGURE 4:**
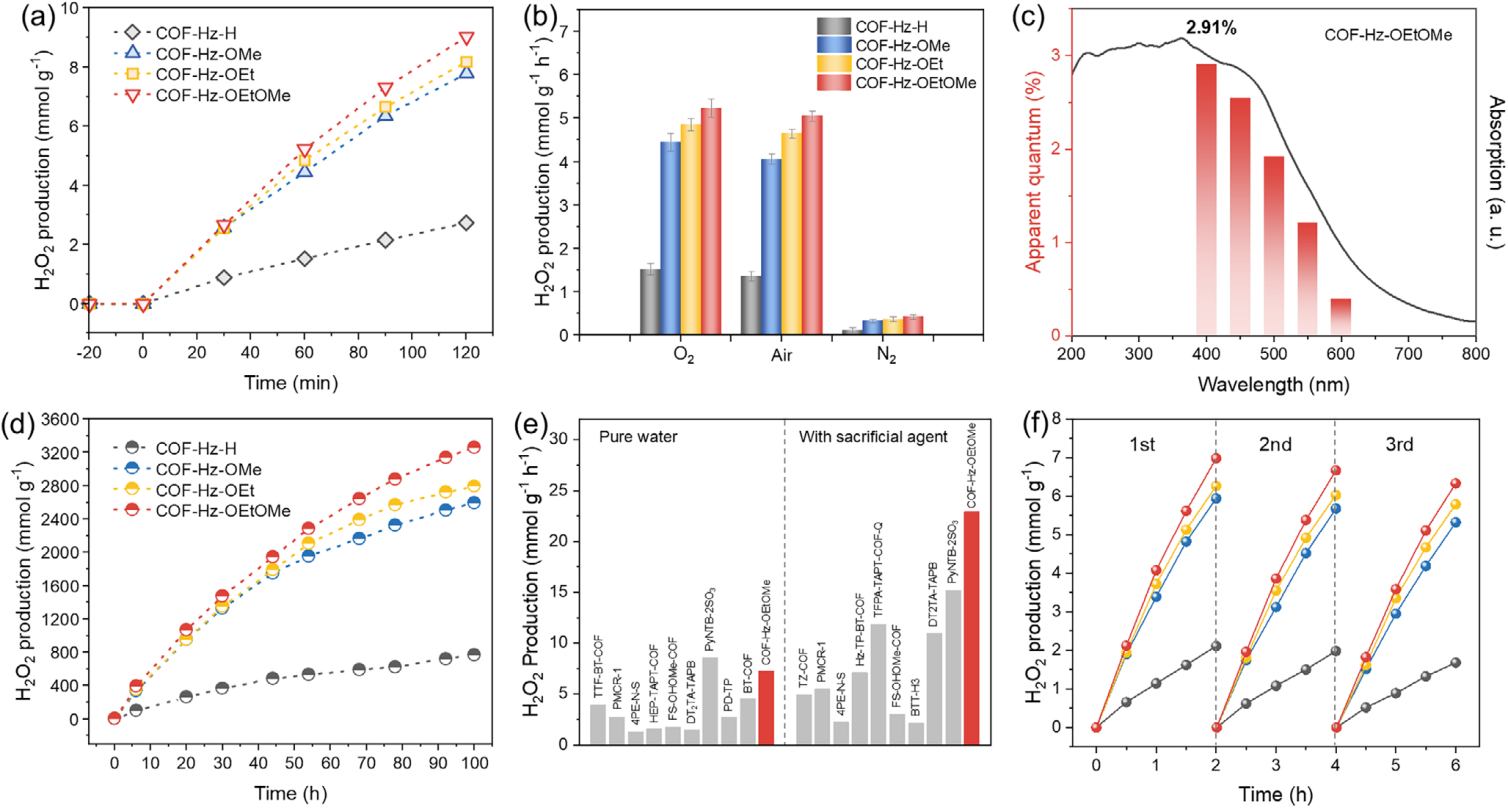
Photocatalytic performance of the COF‐Hzs: (a) H_2_O_2_ accumulation in oxygen‐saturated pure water, (b) rates under oxygen, air, and nitrogen (Error bars represent independent measurements (n = 3)), (c) wavelength‐dependent apparent quantum yields, (d) 100 h operation with 10 vol% benzyl alcohol, (e) literature comparison, and (f) recycle tests. Standard conditions: 5 mg catalyst, 50 mL water, 300 W Xe lamp, 100 mW cm^−2^, and λ ≥ 420 nm unless stated otherwise.

PXRD analysis reveals that the alkoxy‐functionalized COF‐Hz‐OMe, COF‐Hz‐OEt, and COF‐Hz‐OEtOMe exhibit relatively higher crystallinity compared to the pristine COF‐Hz‐H, which possesses a more flexible backbone. Generally, high crystallinity is anticipated to facilitate photocatalytic performance [28]. To further investigate the influence of crystallinity from chemical modification on H_2_O_2_ production, an amorphous counterpart of the optimal COF‐Hz‐OEtOMe was synthesized for comparison (Figure S19). The photocatalytic performance was shown in Figure S20, the H_2_O_2_ evolution rate of the amorphous COF‐Hz‐OEtOMe decreased to 4170 µmol g^−1^ h^−1^, which is lower than that of its crystalline counterpart (5230 µmol g^−1^ h^−1^). Consequently, we conclude that while improved crystallinity contributes to the enhanced activity, its impact is relatively limited (accounting for an approximate 20% difference). The incorporation of alkoxy groups within the pore walls is identified as the primary driver for the substantial surge in photocatalytic performance.

Subsequently, the photocatalytic H_2_O_2_ production performance was evaluated under different gas atmospheres, including pure oxygen, air, and nitrogen. As shown in Figure 4b, the evolution rates of COF‐Hz‐H, COF‐Hz‐OMe, COF‐Hz‐OEt, and COF‐Hz‐OEtOMe under an air atmosphere were 1360, 4060, 4640, and 5050 µmol g^−1^ h^−1^, respectively, which are only slightly lower than those observed under a pure oxygen atmosphere. In contrast, under a nitrogen atmosphere, the corresponding rates dropped to 110, 330, 370, and 420 µmol g^−1^ h^−1^, respectively. These results indicate that H_2_O_2_ can indeed be generated via the water oxidation reaction (WOR) pathway, although its contribution to the total yield is limited compared to the oxygen reduction (ORR) pathway. The impact of catalyst dosage on photocatalytic performance was also investigated by dispersing 1, 3, 5, 10, and 20 mg of the COF‐Hzs in 50 mL of pure water. As shown in Figure S21, the total amount of accumulated H_2_O_2_ increased gradually with the catalyst dosage, but the growth trend slowed down when the dosage reached 20 mg. However, the mass‐normalized evolution rate (yield per gram) decreased as the catalyst dosage increased, which can be attributed to the light scattering effect (or shielding effect) and the limited availability of accessible catalytic sites at higher concentrations [29, 30]. Consequently, the optimal specific activity was achieved with a dosage of 1 mg in 50 mL of water. Specifically, the rates for COF‐Hz‐H, COF‐Hz‐OMe, COF‐Hz‐OEt, and COF‐Hz‐OEtOMe were determined to be 2380, 6620, 6760, and 7240 µmol g^−1^ h^−1^, respectively.

Under monochromatic light irradiation at 400 nm, the apparent quantum yields (AQYs) of COF‐Hz‐OMe, COF‐Hz‐OEt, and COF‐Hz‐OEtOMe were determined to be 2.13%, 2.26%, and 2.91%, respectively (Figures 4c and S22; Tables S3–S6). These values are significantly higher than the pristine COF‐Hz‐H (0.90%). Moreover, the wavelength‐dependent AQY values show a trend consistent with their UV‐Vis absorption spectra, indicating that the reaction is driven by the photo‐excited charge carriers. Furthermore, the solar‐to‐chemical conversion (SCC) efficiency was evaluated using a solar simulator (AM 1.5 G). The results reveal that the SCC values for COF‐Hz‐OMe, COF‐Hz‐OEt, and COF‐Hz‐OEtOMe are 0.48%, 0.53%, and 0.62%, respectively, compared to 0.18% for COF‐Hz‐H.

The selection of an appropriate sacrificial electron donor is pivotal for optimizing charge carrier dynamics. In our evaluation of various alcohols, benzyl alcohol (BA) emerged as a superior sacrificial agent compared to simpler alcohols such as methanol, ethanol, and isopropanol (Figure S23). Notably, the integration of 10% (V/V) BA under an O_2_‐saturated environment triggered a remarkable surge in photocatalytic efficiency. The H_2_O_2_ production rates reached 6880 µmol g^−1^ h^−1^ for COF‐Hz‐H, 16470 µmol g^−1^ h^−1^ for COF‐Hz‐OMe, 17890 µmol g^−1^ h^−1^ for COF‐Hz‐OEt and 22910 µmol g^−1^ h^−1^ for COF‐Hz‐OEtOMe. This enhancement is likely attributed to the lower oxidation potential of BA, which facilitates more efficient hole scavenging and suppresses radiative recombination. To further clarify the coupled redox process, the organic phase after photocatalysis was analyzed by GC‐FID using mesitylene as an internal standard (Figures S24 and S25). Benzaldehyde was identified as the oxidation product of BA. In two independent paired experiments performed under the same photocatalytic conditions, average amounts of 130.4 µmol benzaldehyde and 122.82 µmol H_2_O_2_ were obtained, corresponding to a benzaldehyde/H_2_O_2_ molar ratio of approximately 1.06. Since the oxidation of BA to benzaldehyde and the reduction of O_2_ to H_2_O_2_ are both two‐electron processes, this nearly equimolar relationship supports balanced utilization of photogenerated holes and electrons in the coupled photocatalytic system. In addition, the immiscibility of BA and water creates a biphasic system that spatially separates the oxidation and reduction sites, effectively isolating the produced H_2_O_2_ from the photocatalyst. This partitioned environment prevents backward reactions and optimizes charge utilization, resulting in significantly faster redox kinetics compared to homogeneous systems [10].

The COF‐Hzs demonstrated exceptional long‐term durability, sustained by a continuous 100‐hour photocatalytic run in the 10 vol% BA system without significant loss of activity (Figure 4d). Notably, COF‐Hz‐OEtOMe shows benchmark‐level activity among reported COF‐based photocatalysts using sacrificial agent (Figure 4e and Table S7). However, H_2_O_2_ production rates are highly dependent on experimental conditions, and therefore direct comparisons between different studies should be made with caution.

Beyond their high activity, the COF‐Hz series exhibited excellent stability over three successive reaction cycles (Figure 4f). To assess the structural integrity of the frameworks after catalysis, PXRD, FT‐IR, and XPS analyses were performed on the recovered samples. As shown in Figure S26, the crystalline lattices of the alkoxy‐functionalized COFs remained remarkably stable. While a minor decrease in diffraction intensity occurred because of potential morphological changes or guest molecule adsorption, the core crystallinity was well‐preserved. The FT‐IR spectra of the recovered COFs also retain the characteristic C═N, N─H, and amide C═O vibrations of the hydrazone frameworks, without the appearance of new characteristic bands (Figure S27). Consistently, the high‐resolution C 1s, N 1s, O 1s, and S 2p XPS spectra remain closely comparable to those of the pristine materials, with the characteristic binding‐energy features preserved and no new dominant chemical‐state components observed (Figures S28–S31). These results collectively demonstrate the good structural and chemical stability of the COF‐Hzs under the photocatalytic conditions.

### 2.4 Mechanism of Photocatalytic H_2_O_2_ Production

Given the superior photocatalytic performance of COF‐Hz‐OEtOMe in H_2_O_2_ production, it was selected as a representative model to investigate the reaction mechanism and practical applicability. The pathway for H_2_O_2_ evolution over COF‐Hz‐OEtOMe was thoroughly investigated through a series of control experiments and DFT calculations. Regarding the reduction half‐reaction, no H_2_ was detected by gas chromatography (GC) during the photocatalytic process (Figure S32). This indicates that COF‐Hz‐OEtOMe possesses high selectivity for H_2_O_2_ production via oxygen reduction reaction (ORR), rather than water splitting. Generally, the ORR can proceed via either a stepwise single‐electron pathway or a direct two‐electron pathway, the key distinction lies in the generation of the superoxide radical intermediate (·O_2_^−^), which is absent in the direct pathway. To verify the presence of ·O_2_^−^, electron paramagnetic resonance (EPR) analysis was conducted using 5,5‐dimethyl‐1‐pyrroline N‐oxide (DMPO) as a spin trap. As shown in Figure S33, no signals were detected in the dark. However, characteristic peaks of the DMPO‐·O_2_^−^ adduct appeared after 5 min of visible light irradiation, suggesting that the reaction proceeds via the stepwise ORR pathway involving ·O_2_^−^. To further examine the possible formation of other reactive oxygen species, additional EPR measurements were performed for ·OH using DMPO and for ^1^O_2_ using 2,2,6,6‐tetramethyl‐4‐piperidone (TEMP) as the spin‐trapping agent. No characteristic signals of ·OH or ^1^O_2_ were detected after 5 min of visible‐light irradiation (Figures S34 and S35), indicating that these species are not appreciably generated under the present reaction conditions. This mechanism was further corroborated by scavenger experiments. Upon the addition of 10 mM *p*‐benzoquinone (*p*‐BQ), a typical scavenger for ·O_2_^−^, the production of H_2_O_2_ was significantly suppressed (Figure 5a), confirming the pivotal role of ·O_2_^−^ in the ORR process.

**FIGURE 5:**
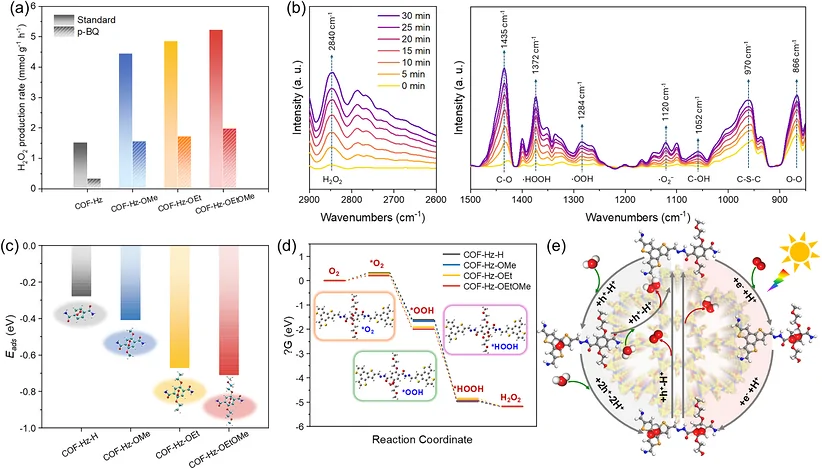
(a) Photocatalytic H_2_O_2_ production rate by COF‐Hzs under the O_2_ atmosphere with *p*‐BQ (10 mM) as a superoxide scavenger. (b) Time‐course in‐situ DRIFTS over COF‐Hz‐OEtOMe under visible light irradiation and an O_2_ atmosphere. (c) The O_2_ adsorption sites and binding energies of COF‐Hzs. (d) Gibbs free energy for photocatalytic H_2_O_2_ production process and different adsorption site of *O_2_, *OOH, *HOOH and H_2_O_2_ on COF‐Hzs. (e) Schematic diagram of the photocatalytic H_2_O_2_ production through the ORR and WOR pathways.

In situ diffuse reflectance infrared Fourier transform spectroscopy (DRIFTS) was employed to provide direct evidence for the reaction intermediates (Figure 5b). The band near 2840 cm^−1^, assigned to the O–H vibration of H_2_O_2_, gradually intensified with irradiation time, confirming the continuous formation of H_2_O_2_ [31, 32]. Meanwhile, the intensity variations of the C─S─C stretching mode from the BTT unit (970 cm^−1^) suggest its active role as a catalytic site [33, 34]. A growing band at 866 cm^−1^ is consistent with O─O bond formation upon O_2_ adsorption, while features at 1120 cm^−1^, 1284 cm^−1^, and 1372 cm^−1^ are assigned to ·O_2_^−^, ·OOH, and ·HOOH intermediates, respectively [35]. These observations strongly corroborate the proposed stepwise two‐electron oxygen reduction pathway. The signal peaks observed at 1052 cm^−1^ and 1435 cm^−1^ can be assigned to C─OH and C─O stretching vibrations of H_2_O oxidation intermediates (*OH), respectively. This provides spectroscopic evidence for the WOR pathway in the photocatalytic system [36].

Subsequently, the oxidation half‐reaction during the photocatalytic process was also investigated. Based on the band structure analysis, water oxidation could potentially yield either H_2_O_2_ or O_2_. The generation of H_2_O_2_ may proceed via a direct 2e^−^ pathway (2H_2_O + 2h^+^ → H_2_O_2_ + 2H^+^) or a stepwise 1e^−^ pathway (H_2_O + h^+^ → ·OH + H^+^, 2·OH → H_2_O_2_), which differ in their energy requirements. Meanwhile, O_2_ evolution typically occurs through a direct 4e^−^ pathway (2H_2_O + 4h^+^ → O_2_ + 4H^+^). However, for the COF‐Hzs, the stepwise 1e^−^ pathway is thermodynamically prohibited due to its high potential requirement (+2.73 V vs. NHE). To clarify the actual pathways, photocatalytic O_2_ evolution experiments were first conducted in a vacuum using 20 mM AgNO_3_ as a sacrificial electron acceptor (Labsolar‐6A, Beijing Perfectlight Technology Co., Ltd.). The O_2_ evolution rates for COF‐Hz‐H, COF‐Hz‐OMe, COF‐Hz‐OEt, and COF‐Hz‐OEtOMe were determined to be 185, 513, 579, and 684 µmol g^−1^ h^−1^, respectively, confirming the existence of the direct 4e^−^ oxidation pathway in the presence of sacrificial agents (Figure S36). Furthermore, under a nitrogen atmosphere, the addition of 10 mM KBrO_3_ as an electron scavenger significantly enhanced the H_2_O_2_ production rates to 460, 1066, 1125, and 1467 µmol g^−1^ h^−1^, respectively (Figure S37). Conversely, the addition of 10% ethanol as a hole scavenger reduced the rates to 34, 210, 224, and 263 µmol g^−1^ h^−1^, respectively. Based on these control experiments, we conclude that the WOR in the presence of sacrificial agents involves both a direct 4e^−^ pathway for O_2_ evolution and a direct 2e^−^ pathway for H_2_O_2_ production. However, the reaction pathway for efficient H_2_O_2_ production without any sacrificial agents remained unclear. Therefore, isotopic labeling experiments were performed using COF‐Hz‐OEtOMe as the photocatalyst and H_2_^18^O as the water source. As shown in Figure S38a, after the photocatalytic reaction, trace amounts of ^18^O_2_ were detected in the gas phase by gas chromatography‐mass spectrometry (GC‐MS), indicating the occurrence of the direct 4e^−^ WOR pathway for oxygen evolution even without sacrificial agents. Subsequently, the reaction vessel was opened, the catalyst was removed by filtration through a 0.22 µm membrane, and MnO_2_ was added to decompose the generated H_2_O_2_. The evolved gas was then analyzed, and ^18^O_2_ was clearly detected (Figure S38b). This result provides definitive evidence that H_2_^18^O serves as the oxygen source for the photocatalytic generation of isotope‐labeled H_2_^18^O_2_ via the WOR pathway. In summary, the photocatalytic water oxidation half‐reaction proceeds via concurrent pathways yielding both O_2_ and H_2_O_2_.

To identify the preferential catalytic centers for oxygen reduction, DFT calculations were first performed to evaluate the possible active sites, including the BTT units, hydrazone linkages, linker‐derived phenyl rings, and alkoxy side chains. The calculations identified the linker‐derived phenyl units as the primary sites for oxygen reduction, whereas the alkoxy side chains mainly regulate the local adsorption environment rather than directly participating in the redox process (Figure S39). The influence of side chain substitution was then evaluated by comparing the O_2_ adsorption energies at the identified reduction sites across the four COF‐Hzs (Figure 5c). The calculated adsorption energies became progressively more favorable from COF‐Hz‐H (−0.28 eV) to COF‐Hz‐OMe (−0.41 eV), COF‐Hz‐OEt (−0.67 eV), and COF‐Hz‐OEtOMe (−0.71 eV), indicating increasingly stronger oxygen affinity with extended alkoxy side chains. These results suggest that the alkoxy‐functionalized nanochannels promote oxygen activation by creating a more favorable local adsorption environment adjacent to the catalytic sites, rather than by acting as catalytic centers themselves.

Gibbs free energy (ΔG) profiles for the four reaction steps of the ORR pathway (O_2_→*O_2_→*OOH→*HOOH→H_2_O_2_, where * represents the catalytic site) were then calculated (Figures 5d and S40). The adsorbed O_2_ molecule is first activated into the activated state (*O_2_), which then undergoes a hydrogenation process to generate the *OOH intermediate, eventually leading to the production of H_2_O_2_. Based on the ΔG profiles, the initial activation of O_2_ (O_2_→*O_2_) is identified as the rate‐determining step (RDS). Compared to pristine COF‐Hz‐H (0.31 eV), COF‐Hz‐OMe (0.29 eV), and COF‐Hz‐OEt (0.28 eV), COF‐Hz‐OEtOMe exhibited the lowest energy barriers for this RDS (0.21 eV). To fundamentally unravel the intrinsic origin of this enhanced performance, the surface electrostatic potential (ESP) of the four COFs was mapped (Figure S41). Electrostatic‐potential maps show increasingly negative regions (highlighted in deep red) near the oxygen‐rich pore wall, consistent with stabilization of adsorbed oxygen species and facilitated electron transfer into oxygen‐derived orbitals. This enriched electronic microenvironment serves a dual purpose: (i) it facilitates more efficient electron injection from the catalyst to the π* orbital of O_2_, thereby effectively promoting its activation; and (ii) it stabilizes the adsorbed oxygen species through enhanced local dipole‐dipole interactions. To further distinguish the effect of side‐chain length from that of the additional ether oxygen in COF‐Hz‐OEtOMe, a butoxy‐functionalized control COF (COF‐Hz‐OBu) was synthesized (Figures S42 and S43), in which the ether oxygen in the OEtOMe side chain was replaced by a carbon atom while maintaining the same number of heavy atoms. COF‐Hz‐OBu exhibited a lower H_2_O_2_ production rate of 4593 µmol g^−1^ h^−1^ than COF‐Hz‐OEtOMe (5230 µmol g^−1^ h^−1^) under identical conditions (Figure S44). This result indicates that the enhanced activity of COF‐Hz‐OEtOMe cannot be attributed solely to the increased side‐chain length and supports a contribution from the additional ether oxygen to the local pore microenvironment. Overall, these experimental and theoretical results support that the oxygen‐rich pore microenvironment of COF‐Hz‐OEtOMe contributes to its enhanced O_2_ activation and photocatalytic activity. These theoretical findings are highly consistent with experimental observations, confirming that the ‐OEtOMe modification is key to enhanced photoactivity in photocatalytic H_2_O_2_ production.

Based on these results, a spatially separated redox mechanism for H_2_O_2_ photosynthesis over the COF‐Hzs is proposed (Figure 5e). The conjugated framework spatially separates photogenerated electrons and holes, with the linker‐derived phenyl units serving as the primary sites for oxygen reduction and the electron‐rich BTT nodes driving water oxidation. Meanwhile, the alkoxy‐functionalized nanochannels enrich water and oxygen around the corresponding redox sites and lower the energetic barrier for oxygen activation. These complementary functions illustrate how side chain‐defined nanochannels work synergistically with the conjugated framework to couple charge dynamics with reactant transport, thereby promoting efficient H_2_O_2_ photosynthesis across the COF‐Hz series.

### 2.5 Solar‐Driven H_2_O_2_ Production and Life Cycle Assessment

To determine whether COF‐Hz‐OEtOMe can maintain its H_2_O_2_ photocatalytic production performance in real water samples, the photocatalytic H_2_O_2_ production rates of COF‐Hz‐OEtOMe in tap water, river water, and sea water were also obtained (Figure 6a). Similar to H_2_O_2_ photosynthesis in pure water, the efficiencies were also high in all three tested real water samples. Specifically, after 120 min of visible light irradiation, the H_2_O_2_ photosynthesis rate by COF‐Hz‐OEtOMe in tap water, river water, and sea water reached 5130, 5080, and 4860 µmol g^−1^ h^−1^, respectively. These results clearly demonstrate that COF‐Hz‐OEtOMe can effectively produce H_2_O_2_ in a variety of real water samples with complex water matrix conditions.

**FIGURE 6:**
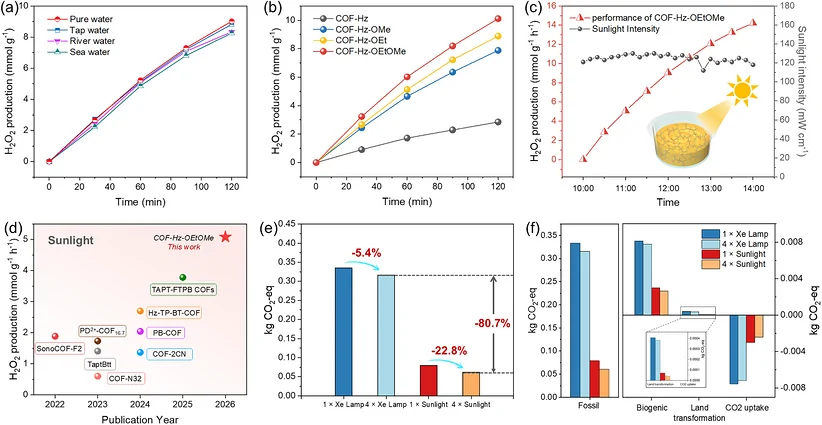
(a) Photocatalytic H_2_O_2_ yield by COF‐Hz‐OEtOMe in pure water, tap water, river water, and sea water under visible‐light irradiation. (b) Photocatalytic H_2_O_2_ production by COF‐Hzs under a solar simulator (AM 1.5 G). (c) Photocatalytic H_2_O_2_ production by COF‐Hz‐OEtOMe under natural sunlight on a sunny day from tap water and air. (d) Comparison of photocatalytic H_2_O_2_ production performance with COF‐based photocatalysts driven by natural sunlight and simulated sunlight (AM 1.5 G). (e) Total greenhouse gas (GHG) emissions (in kg CO_2_‐eq) comparison of 1 mg H_2_O_2_ production under laboratory photocatalysis (Xe lamp) and natural sunlight irradiation. (f) Contribution analysis of different emission sources to the total GWP. Negative values in the “CO_2_ uptake” category represent carbon sequestration or environmental credit. Values for land transformation and CO_2_ uptake are enlarged in the inset for better visibility. *Note*: 1 × refers to baseline synthesis of COF; 4 × refers to fourfold‐scale synthesis; All results are calculated per functional unit of 1 mg H_2_O_2_.

Encouraged by the remarkable performance of COF‐Hzs in lab‐based environments under xenon lamp irradiation even in air and tap water, we extended our investigation to outdoor scale‐up experiments using natural sunlight. Preliminary tests under AM 1.5G irradiation yielded a peak H_2_O_2_ rate of 1720 µmol g^−1^ h^−1^, 4650 µmol g^−1^ h^−1^, 5130 µmol g^−1^ h^−1^ and 6020 µmol g^−1^ h^−1^ for COF‐Hz‐H, COF‐Hz‐OMe, COF‐Hz‐OEt, and COF‐Hz‐OEtOMe, respectively (Figure 6b). Outdoor operation was then tested with 100 mg COF‐Hz‐OEtOMe in 1 L of tap water under air and natural sunlight. At 27∼36°C and 112∼130 mW cm^−2^ irradiance, the system reached an initial mass‐normalized rate of 5080 µmol g^−1^ h^−1^ and accumulated 1.42 mM hydrogen peroxide after 4 h (Figures 6c,d and S45), satisfying the requisite concentration levels for water purification tasks [37]. These results significantly exceed most previously reported benchmarks driven by both natural sunlight and simulated sunlight (AM 1.5 G). (Figure 6d) [34, 38, 39, 40, 41, 42, 43, 44].

The environmental impacts of photocatalytic H_2_O_2_ were evaluated using LCAs by the GWP100 (including carbon dioxide absorption) methodology published by the Intergovernmental Panel on Climate Change (IPCC) in 2021 [45]. This metric expresses greenhouse gas emissions in terms of carbon dioxide equivalents over a 100‐year time horizon, thereby enabling consistent comparisons between different emission sources.

The LCA results for the production of 1 mg of H_2_O_2_ indicate that the photocatalytic process is predominantly influenced by fossil energy contributions, which account for the largest proportion across all scenarios. As illustrated in Figure 6e; Tables S8 and S9, under Xe lamp irradiation, increasing the catalyst synthesis scale fourfold reduces the calculated impact slightly from 0.334 to 0.316 kg CO_2_‐eq per functional unit (−5.4%), indicating a modest scale‐up benefit within the defined system boundary. In contrast, under natural sunlight, a more pronounced reduction is observed, with the impact decreasing from 0.079 to 0.061 kg CO_2_‐eq (−22.8%). Notably, switching from Xe lamp irradiation to natural sunlight leads to an emission reduction of up to 80.7% and a significantly lower environmental impact, primarily due to the elimination of artificial energy inputs. Among the various impact categories, contributions from biogenic sources and land transformation are minimal, while CO_2_ uptake provides a small negative contribution, partially offsetting total emissions (Figure 6f). Among all evaluated conditions, the lowest environmental impact (0.0611 kg CO_2_‐eq) is achieved under natural sunlight with fourfold‐scaled catalyst synthesis, highlighting that the combination of enhanced catalytic efficiency and renewable energy input represents the most sustainable pathway for photocatalytic H_2_O_2_ production.

## 3 Conclusion

In summary, a series of hydrazone‐linked COFs with systematically varied pore‐wall side chains were developed for photocatalytic H_2_O_2_ production. Alkoxy substitution modulates the nanochannel microenvironment by tuning accessible porosity, pore polarity, water and oxygen adsorption, and charge dynamics while preserving the same framework topology. Although COF‐Hz‐OEtOMe possesses a lower BET surface area than COF‐Hz‐OMe, it exhibits the highest photocatalytic activity, highlighting that nanochannel functionality rather than surface area governs performance. Experimental and theoretical studies reveal a division of function in which the conjugated framework governs charge dynamics, whereas the alkoxy‐functionalized nanochannels enrich reactants and facilitate oxygen activation. Together with the excellent performance under practical conditions, these findings establish pore‐wall side chains as functional elements that couple charge dynamics with reactant transport, providing a general design principle for porous photocatalysts.

## Author Contributions

**Ruixue Sun**: conceptualization, methodology, data curation, writing – original draft, writing – review and editing, supervision, validation, investigation, formal analysis. **Shaoyang Gao**: software, visualization, validation, investigation, writing – original draft. **Luqiao Feng**: software, validation, writing – original draft, data curation. **Peixuan Xie**: data curation, investigation, validation. **Wenyu Shen**: methodology, data curation, validation. **Nikita Rog**: methodology, validation, data curation. **Marwan Mohammed**: methodology, validation, data curation. **Qi Huang**: conceptualization, methodology, writing – review and editing, validation. **Giulio Santori**: methodology, validation, data curation, writing – review and editing. **Xianfeng Fan**: writing – review and editing, methodology, project administration, funding acquisition. **Bien Tan**: conceptualization, writing – review and editing, supervision, funding acquisition, project administration, formal analysis. **Cher Hon Lau**: conceptualization, methodology, supervision, resources, project administration, funding acquisition, validation, writing – original draft, writing – review and editing, formal analysis.

## Conflicts of Interest

The authors declare no conflicts of interest.

## Supporting information




**Supporting File**: anie74636‐sup‐0001‐SuppMat.docx.

## Data Availability

The data that support the findings of this study are available from the corresponding author upon reasonable request.
