# Supplementary material for: Modulating Covalent Organic Framework Nanochannel Microenvironments for Sustainable Hydrogen Peroxide Photosynthesis
Source: Angew Chem Int Ed Engl. 2026 Sep 4;65(44):e7792190. doi: 10.1002/anie.7792190 (PMC13703877; doi:10.1002/anie.7792190)
Supplement: Supplementary file 1 [file ANIE-65-e7792190-s001.docx]

Supporting Information

Modulating Covalent Organic Framework Nanochannel Microenvironments for Sustainable Hydrogen Peroxide Photosynthesis

Ruixue Sun^1,^*, Shaoyang Gao^1^, Luqiao Feng^1^, Peixuan Xie^2^, Wenyu Shen^1^, Nikita Rog^1^, Marwan Mohammed^1^, Qi Huang^1^, Giulio Santori^1^, Xianfeng Fan^1^, Bien Tan^2,^*, and Cher Hon Lau^1,^*

Table of Contents:

Section 1. Materials and Characterization

Section 2. Synthetic procedure of covalent organic frameworks

Section 3. H_2_O_2_ photosynthesis experiment

Section 4. Figures S1-S45

Section 5. Tables S1-S9

**Section 1. Materials and Characterization**

**Materials**

Benzo[1,2-b:3,4-b':5,6-b'']trithiophene-2,5,8-tricarbaldehyde, terephthalohydrazide, 2,5-dimethoxyterephthalohydrazide, 2,5-diethoxyterephthalohydrazide, and 2,5-bis(2-methoxyethoxy)terephthalohydrazide were purchased from Merck. All purchased chemicals were utilized without further purification.

**Characterization**

Fourier-transform infrared (FTIR) spectra were recorded on a Shimadzu IRTracer-100 spectrometer over the range of 4000~500 cm⁻¹. Solid-state ^13^C cross-polarization magic angle spinning (CP/MAS NMR) spectra were recorded on a 500 MHz Bruker AVANCE NEO spectrometer. Elemental analysis (EA) measurements were performed on a Thermo Fisher FlashSmart 2000 Organic Elemental Analyser with a MAS Plus autosampler for solid samples. High-resolution scanning electron microscopy (SEM) images were obtained using a Cryo FIB-SEM system to analyze the morphology of COF-Hzs. The powder samples were mounted on carbon adhesive tabs, which contributed to the background O signal in the EDS mapping. Thermogravimetric analysis (TGA) was performed on a TA Instruments Trios V5 system under a nitrogen atmosphere at a heating rate of 10 °C min⁻¹ from room temperature to 800 °C. Powder X-ray diffraction (PXRD) patterns were recorded on a Bruker D8 Advance diffractometer equipped with a Sol-X energy-dispersive detector. UV-Vis absorption spectra of the polymers were measured on a Shimadzu UV-2600 UV-Vis spectrometer by measuring the reflectance of powders in the solid state. The transient-state photoluminescence decay profiles were obtained on an QuantaMaster 8000 instrument (HORIBA, Canada) with an excitation wavelength at 340 nm. Gas chromatography (GC) analysis of the benzyl alcohol oxidation products was performed using a Shimadzu GC-2020 Plus gas chromatograph equipped with an AOC-20i auto injector and a flame ionization detector (FID).

**Nitrogen sorption analysis**

N_2_ adsorption isotherms (77 K) and pore size distributions were measured using a Quadrasorb Evo instrument (Quantachrome) surface area and porosity analyzer. Samples were degassed at 100°C for 12 h under vacuum (10^−5^ bar) before the analysis. The surface areas were calculated based on nitrogen adsorption isotherms by Brunauer-Emmett-Teller (BET) analysis. Pore size distributions were calculated by non-local density functional theory (NLDFT) method using the adsorption branch. The NLDFT output was given as half pore width, and the values were converted to full pore width for presentation.

**Electrochemical measurements**

Electrochemical measurements were performed using three electrode systems on a CHI 760E electrochemical workstation. The transient photocurrent, Electrochemical impedance spectra and the Mott-Schottky of COF-Hzs were conducted under 300W Xe light irradiation, 0.2 M Na_2_SO_4_ solution was selected as supporting electrolyte and the pH value is 7. The samples were prepared by mixing polymer powder (2 mg) with isopropanol (0.3 mL) containing 5 wt% Nafion, and then were sonicated 10 min. 200 μL sample was taken and deposited on ITO with an area of 1 cm^2^. The conversion of the Ag/AgCl electrode reference potential to a normal hydrogen electrode (NHE) was calculated using the following equation.

E(NHE) = E(Ag/AgCl) + E^0^(Ag/AgCl), (E^0^(Ag/AgCl) = 0.197 V)

For n-type semiconductors, the flat-band potential is generally close to the conduction-band/LUMO edge. Therefore, the LUMO potentials were approximated from the measured flat-band potentials.

E_LUMO_ ≈ E_fb_

**Electron paramagnetic resonance (EPR)**

Electron paramagnetic resonance (EPR) measurements of COF-Hzs were performed at room temperature. DMPO was used as a spin-trapping agent for the detection of ·O_2_⁻ and ·OH. For ·O_2_⁻ detection, 4 mg of COF-Hzs was dispersed in 2 mL of dried DMSO containing 100 mM DMPO, and the EPR spectra were recorded before and after visible-light irradiation for 3 min. For ·OH detection, 2 mg of COF-Hzs was dispersed in 500 μL of water containing 0.1 mmol DMPO. The dispersion was purged with O_2_ for 5 min before measurement, and the EPR spectra were recorded under dark conditions and after visible-light irradiation for 5 min. For singlet oxygen (^1^O_2_) detection, 2 mg of COF-Hzs was dispersed in 500 μL of water containing 0.1 mmol TEMP as the spin-trapping agent. After purging with O_2_ for 5 min, the EPR spectra were recorded under dark conditions and after visible-light irradiation for 5 min.

**Temperature-dependent photoluminescence (PL) spectra**

The temperature-dependent photoluminescence (PL) spectra were recorded using a FLS980 spectrometer in the solid state. The measurements were conducted at different temperatures, *i. e.*, 80 K, 150 K, 200 K, 250 K, and 298 K.

**In situ diffuse reflectance infrared Fourier transform spectroscopy (****DRIFTS)**

In situ diffuse reflectance infrared Fourier transform spectroscopy (DRIFTS) measurements were performed using a Bruker VERTEX 80v FT-IR spectrometer equipped with an in situ diffuse-reflectance reaction cell. COF-Hz-OEtOMe was placed in the reaction cell and pretreated before collection of the background spectrum. H_2_O and O_2_ were subsequently introduced under dark conditions, followed by visible-light irradiation. The in situ DRIFTS spectra were recorded at 5 min intervals over a total irradiation time of 30 min. All spectroscopic measurements were collected with a resolution of 4 cm^−1^ and at least 128 scans.

**Apparent quantum yield (AQY) measurement**

The AQY was measured using the following conditions: 50 mg of COF-Hzs was fully dispersed in a Pyrex glass reaction cell containing 50 mL deionized water, and band pass filters (400 nm, 450 nm, 500 nm, 550 nm, 600 nm). The light intensity at 400 nm, 450 nm, 500 nm, 550 nm and 600 nm was 17.4, 17.9, 22.3, 24.9 and 21.6 mW cm^−2^, respectively. The reaction system temperature was maintained at 20°C by a flow of cooling water. The final AQY at a certain light irradiation wavelength was calculated according to the equation:

$$\mathrm{AQY}\left( \% \right)=\frac{2CN_{A}}{\mathrm{SP}t\lambda/hc}\times100\%$$

where C is the H_2_O_2_ produced (mol) per hour; N_A_ is the Avogadro constant (6.023 × 10^23^/mol), h is the Planck constant (6.626 × 10^−34^ J·s), S is irradiation area (19.63 cm^2^, d = 5 cm); c is vacuum light velocity (3 × 10^8^ m/s), λ is the monochromatic light wavelength (m), t is the light irradiation time (3600 s) and P is the incident monochromatic light intensity (W·cm^−2^). The photocatalytic rates are not proportional to the photocatalyst mass because light absorption reaches saturation at some point. AQY should accordingly be measured when the amount of photocatalyst is sufficient, and the incident light is effectively absorbed by the photocatalysts. The equation does not include the parameter: catalyst amount, which means the AQY value would vary with catalyst amount. It’s worth noting that the AQY reflects the catalyst’s performance around a specific wavelength, and the value is highly dependent on specific experimental parameters such as the intensity and wavelength of the incident monochromatic light, as well as the illuminated area. These parameters can vary across different laboratories and different devices, introducing potential sources of error in the determination of AQY values.

**The solar-to-chemical energy conversion (SCC) efficiency measurement**

The 50 mg photocatalyst was well dispersed in a quartz vessel containing 50 mL deionized water and then degassed by O_2_ bubbling for 10 min before sealed in vessel. A Xenon solar simulator was used as the light source (AM 1.5). The reaction temperature was kept at 20°C. The SCC efficiency was calculated according to the following equation:

$$SCC \left( \% \right)=\frac{\left[ \Delta G for H_{2}O_{2} generation \left( \frac{J}{mol} \right) \right]\times\left[ H_{2}O_{2} produced \left( mol \right) \right]}{\left[ total input energy \left( W \right) \right]\times\left[ reaction times \left( s \right) \right]} \times100$$

Where the free energy (∆G) for H_2_O_2_ formation is 117 kJ mol^−1^, the irradiance of the spectrum is 1000 W m^−2^ and the irradiated area is 3.14 × 10^−4^ m^2^.

**DFT calculations**

The structure of four COFs were modeled based on the PXDR results, and the structure was optimized by CP2K with a cutoff energy of 550 Ry under the level of PBE/ TZVP-MOLOPT-GTH/D3(BJ), and the k point was set as 1,1,10.^[1, 2, 3]^ And then HOMO-LUMO and ESP were calculated based on optimized structure and were plotted using Multiwfn and VMD.^[4, 5]^ As for the binding energy calculation of COF and H_2_O and O_2_, the structure was optimized under PBE/DZVP-MOLOPT-SR-GTH/D3(BJ), then energy was calculated under PBE/ TZVP-MOLOPT-GTH/D3(BJ). The binding energy (BE) was calculated using:

$$BE=E_{COF-molecule}-E_{\mathrm{COF}}-E_{\mathrm{molecule}}$$

where E(COF-molecule) is the energy of the geometry for molecule adsorbed on COF, and E(COF) and E(molecule) are the energies of corresponding COF and molecule.

All the calculations in photocatalytic H_2_O_2_ production process were performed with Gaussian 16 (Revision A.03) under the level of B3LYP/def2tzvp/ GD3BJ.^[6, 7, 8, 9, 10]^ All optimized geometries were validated by frequency analysis, and the Gibbs free energy (ΔG) was calculated using:

$$\Delta G=\sum G \left( products \right)-\sum G \left( reactants \right)$$

where G = E_elec_ + G_corr_, and G_corr_ is the thermal correction to Gibbs free energy provided by Shermo ^[11]^.

**Life cycle assessment (LCA)**

The life cycle assessment of COF-Hz-OEtOMe hydrogen peroxide production lays the groundwork for the wider application of this technology and contributes to the optimization of the production process. Furthermore, LCA data on global electricity consumption was utilized during the modelling process to better categories the energy dynamics associated with electricity use.

This life cycle assessment was conducted in accordance with ISO 14040 and ISO 14044 standards. The calculations were performed using the licensed SimaPro 10.3 Education Edition software. The environmental performance of the system was systematically evaluated through the application of appropriate environmental impact assessment methods.

Using a life cycle assessment (LCA) by the GWP100 (including carbon dioxide absorption) methodology published by the Intergovernmental Panel on Climate Change (IPCC) in 2021, the climate impacts associated with hydrogen peroxide production were systematically quantified. This metric expresses greenhouse gas emissions in terms of carbon dioxide equivalents over a 100-year time horizon, thereby enabling consistent comparisons between different emission sources.

Raw Materials：

Benzo[1,2-b:3,4-b':5,6-b'']trithiophene-2,5,8-tricarbaldehyde (105.6 mg, 0.32 mmol), 2,5-bis(2-methoxyethoxy)terephthalohydrazide (165.6 mg, 0.48 mmol), DMSO (8 mL), 6 M acetic acid (2.4 mL).

Electric power: 650W* 72 h = 46.8 kWh (electricity required for reaction heating for 72h).

Oven powder: 450W * 8 h = 3.6 kWh (electricity required for drying for 8 h).

Reaction Product: COF-Hz-OEtOMe 192 mg

Synthesis Details：

Benzo[1,2-b:3,4-b':5,6-b'']trithiophene-2,5,8-tricarbaldehyde (105.6 mg, 0.32 mmol) and 2,5-bis(2-methoxyethoxy)terephthalohydrazide (165.6 mg, 0.48 mmol) were taken in a 25 mL Pyrex tube, and dissolved in DMSO (8 mL) along with the addition of 6 M acetic acid (2.4 mL). The mixture in the Pyrex tube was sonicated for 10 min. The sealed tube was heated at 120°C for 72 h. After cooling to room temperature, the powder was filtered and washed with acetone (200 mL), tetrahydrofuran (200 mL), and ethanol (200 mL). The filtered solid was placed in a vacuum oven and dried at 60 °C for 8 hours to obtain a deep yellow powder sample. (192 mg, 76.0% yield).

Photocatalytic Process in the Lab：

Raw Material：

O_2_: 60 mL/min* 10 min = 600 mL

Xe Light: 300 W*1 h = 0.3 kWh

COF: 5 mg

Pure H_2_O: 50 g (50 mL)

Circulating water (Tap water): 1 L

Reaction product: Product H_2_O_2_ (0.86 mg, 25.25 μmol)

Photocatalytic Process under sunlight：

Raw Material：COFs 10 mg, Tap water: 100 g (100 mL), air (free)

Product: H_2_O_2_ (1 L) concentration 508 μM corresponding to a total of 1 L * 508 μM = 508 μmol (17.3 mg).

**Section 2. Synthetic procedure of covalent organic frameworks**

**Synthesis of COF-Hz-H^[12]^**

Benzo[1,2-b:3,4-b':5,6-b'']trithiophene-2,5,8-tricarbaldehyde (105.6 mg, 0.32 mmol) and terephthalohydrazide (93.2 mg, 0.48 mmol) were taken in a 10 mL Pyrex tube, and dissolved in DMSO (2 mL) and DMAC (6 mL) along with the addition of 6 M acetic acid (2.8 mL). The mixture in the Pyrex tube was sonicated for 10 min. The sealed tube was heated at 120°C for 72 h. After cooling to room temperature, the powder was filtered and washed with acetone, tetrahydrofuran, and ethanol. The filtered solid was placed in a vacuum oven and dried at 60 °C for 24 hours to obtain a deep yellow powder sample. (140 mg, 77.1% yield).

**Synthesis of COF-Hz-OMe**

Benzo[1,2-b:3,4-b':5,6-b'']trithiophene-2,5,8-tricarbaldehyde (105.6 mg, 0.32 mmol) and 2,5-dimethoxyterephthalohydrazide (122.0 mg, 0.48 mmol) were taken in a 10 mL Pyrex tube, and dissolved in DMSO (8 mL) along with the addition of 6 M acetic acid (2.4 mL). The mixture in the Pyrex tube was sonicated for 10 min. The sealed tube was heated at 120°C for 72 h. After cooling to room temperature, the powder was filtered and washed with acetone, tetrahydrofuran, and ethanol. The filtered solid was placed in a vacuum oven and dried at 60 °C for 24 hours to obtain a deep yellow powder sample. (164 mg, 78.0% yield).

**Synthesis of COF-Hz-OEt**

Benzo[1,2-b:3,4-b':5,6-b'']trithiophene-2,5,8-tricarbaldehyde (105.6 mg, 0.32 mmol) and 2,5-diethoxyterephthalohydrazide (135.6 mg, 0.48 mmol) were taken in a 10 mL Pyrex tube, and dissolved in DMSO (8 mL) along with the addition of 6 M acetic acid (2.4 mL). The mixture in the Pyrex tube was sonicated for 10 min. The sealed tube was heated at 120°C for 72 h. After cooling to room temperature, the powder was filtered and washed with acetone, tetrahydrofuran, and ethanol. The filtered solid was placed in a vacuum oven and dried at 60 °C for 24 hours to obtain a deep yellow powder sample. (176 mg, 78.6% yield).

**Synthesis of COF-Hz-OEtOMe**

Benzo[1,2-b:3,4-b':5,6-b'']trithiophene-2,5,8-tricarbaldehyde (105.6 mg, 0.32 mmol) and 2,5-bis(2-methoxyethoxy)terephthalohydrazide (164.4 mg, 0.48 mmol) were taken in a 25 mL Pyrex tube, and dissolved in DMSO (8 mL) along with the addition of 6 M acetic acid (2.4 mL). The mixture in the Pyrex tube was sonicated for 10 min. The sealed tube was heated at 120°C for 72 h. After cooling to room temperature, the powder was filtered and washed with acetone, tetrahydrofuran, and ethanol. The filtered solid was placed in a vacuum oven and dried at 60 °C for 24 hours to obtain a deep yellow powder sample. (192 mg, 76.0% yield).

**Synthesis of COF-Hz-OBu**

Benzo[1,2-b:3,4-b':5,6-b'']trithiophene-2,5,8-tricarbaldehyde (105.6 mg, 0.32 mmol) and 2,5-bis(n-butoxy)terephthalohydrazide (162.4 mg, 0.48 mmol) were taken in a 25 mL Pyrex tube, and dissolved in DMSO (8 mL) along with the addition of 6 M acetic acid (2.4 mL). The mixture in the Pyrex tube was sonicated for 10 min. The sealed tube was heated at 120°C for 72 h. After cooling to room temperature, the powder was filtered and washed with acetone, tetrahydrofuran, and ethanol. The filtered solid was placed in a vacuum oven and dried at 60 °C for 24 hours to obtain a deep yellow powder sample. (188 mg, 75% yield).

**Synthesis of Amorphous COF-Hz-OEtOMe**

Benzo[1,2-b:3,4-b':5,6-b'']trithiophene-2,5,8-tricarbaldehyde (105.6 mg, 0.32 mmol) and 2,5-bis(2-methoxyethoxy)terephthalohydrazide (164.4 mg, 0.48 mmol) were taken in a 25 mL Pyrex tube, and dissolved in mesitylene (4 mL) and 1,4-dioxane (4 mL) along with the addition of 6 M acetic acid (0.8 mL). The mixture in the Pyrex tube was sonicated for 10 min. The sealed tube was heated at 120°C for 72 h. After cooling to room temperature, the powder was filtered and washed with acetone, tetrahydrofuran, and ethanol. The filtered solid was placed in a vacuum oven and dried at 60 °C for 24 hours to obtain a deep yellow powder sample. (180 mg, 71.1% yield).

**Section 3. H_2_O_2_ photosynthesis experiment**

**H_2_O_2_ photosynthesis under laboratory** **conditions**

The photocatalyst COF-Hzs (5 mg) was fully dispersed in 50 mL of pure water using ultrasonic treatment for 10 minutes. The resulting mixture was transferred to a reaction flask and illuminated using a high power Xenon light source (PLS-SXE300E, Beijing Perfectlight Technology Co., Ltd.) equipped with a filter (λ ≥ 420 nm / AM 1.5 G) and stirring under O_2_, air or N_2_ atmosphere. Circulating cooling water was employed to keep the photocatalytic reaction temperature at 20°C. Following the reaction, a 1 mL sample of the solution was extracted using an injection syringe and subsequently filtered through a 0.2 μm Millipore filter to remove the photocatalyst. H_2_O_2_ production was determined using the iodometry method and the amount of H_2_O_2_ was calculated based on the standard curve. The average rate of photocatalytic H_2_O_2_ production was calculated based on the amount of H_2_O_2_ within the first 1 h.

**H_2_O_2_ photosynthesis under natural sunlight**

The photocatalyst COF-Hz-OEtOMe (100 mg) was fully dispersed in 1 L of tap water using ultrasonic treatment for 30 minutes. The resulting mixture was transferred to a larger cylindrical glass reactor with a diameter of 18 cm and illuminated under sunlight on a sunny day, while stirring under an air atmosphere. The sunlight intensity and photocatalytic reaction temperature were monitored by a thermometer every 10 minutes. Following the reaction, a 1 mL sample of the solution was extracted using an injection syringe and subsequently filtered through a 0.2 μm Millipore filter to remove the photocatalyst. H_2_O_2_ production was determined using the iodometry method and the amount of H_2_O_2_ was calculated based on the standard curve.

**H_2_O_2_ detection methods**

The amount of H_2_O_2_ was determined by iodometry using UV-Vis spectrophotometer. A solution was prepared by adding 1 mL of 0.1 mol L^−1^ potassium hydrogen phthalate (C_8_H_5_KO_4_) aqueous solution and 1 mL of 0.4 mol L^−1^ potassium iodide (KI) aqueous solution to the obtained solution (1 mL), which was then allowed to stand for 30 min. The H_2_O_2_ molecules reacted with iodine ions (I^−^) in an acidic environment to produce triiodide ions (I_3_^−^) (H_2_O_2_ + 3I^−^ + 2H+ → I_3_^−^ + 2H_2_O), which exhibited strong absorption near 350 nm. The absorbance at 350 nm was determined using a Shimadzu UV-2600 UV-Vis spectrometer, from which the amount of H_2_O_2_ generated in each reaction was calculated.

The linear relationship between the amount of H_2_O_2_ in 1 mL sample and the absorption intensity was established as follows.

$$y=8.07\times x$$

Where y represents the absorbance intensity at 350 nm, and x denotes the amount (μmol) of H_2_O_2_ in a 1 mL sample.

**Section 4. Figures S1-S45**

**Figure S1.** PXRD patterns of monomers and COF-Hzs.

**Figure S2.** Top views of the simulated structures for the COF-Hzs in both AA (top) and AB (bottom) stacking modes.

**Figure S3.** Simulated PXRD of AA and AB stacking patterns for COF-Hzs.

**Figure S4.** HRTEM images of COF-Hzs.

**Figure S5.** FT-IR spectra of COF-Hzs.

**Figure S6.** High-resolution XPS spectra of COF-Hz-H, COF-Hz-OMe, COF-Hz-OEt, and COF-Hz-OEtOMe: (a) C 1s, (b) N 1s, (c) O 1s, and (d) S 2p regions.

**Figure S7.** PXRD patterns of COF-Hzs before and after immersion in DMF, THF, methanol, and acetone for three days.

**Figure S8.** FT-IR spectra of COF-Hzs before and after immersion in DMF, THF, methanol, and acetone for three days.

**Figure S9.** Pore size distributions of COF-Hzs.

**Figure S10.** SEM image and EDS mapping of COF-Hz-H.

**Figure S11.** SEM image and EDS mapping of COF-Hz-OMe.

**Figure S12.** SEM image and EDS mapping of COF-Hz-OEt.

**Figure S13.** TGA curve of COF-Hzs.

**Figure S14.** Temperature-dependent photoluminescence spectra of COF-Hz-H, COF-Hz-OMe, COF-Hz-OEt.

**Figure S15.** Time-dependent photoluminescence (PL) decay spectra of COF-Hz-H, COF-Hz-OMe and COF-Hz-OEt.

**Figure S16.** The Tauc-plots of COF-Hzs.

**Figure S17.** Mott-Schottky plots of COF-Hzs.

**Figure S18.** Photocatalytic H_2_O_2_ production toward the monomers and COF-Hzs. Photocatalytic conditions: 5 mg photocatalyst, 50 mL of pure water, O_2_ atmosphere.

**Figure S19.** PXRD patterns of crystalline and amorphous COF-Hz-OEtOMe synthesized in different solvents: crystalline sample from DMSO and amorphous sample from mesitylene/dioxane = 1/1.

**Figure S20.** Photocatalytic performance of crystalline and amorphous COF-Hz-OEtOMe.

**Figure S21.** Photocatalytic H_2_O_2_ production of COF-Hzs with different dosage in the same photocatalytic condition. Photocatalytic conditions: 50 mL of pure water, O_2_ atmosphere.

**Figure S22.** AQY of H_2_O_2_ photosynthesis of COF-Hz-H, COF-Hz-OMe and COF-Hz-OEt at various incident light wavelengths. Photocatalytic conditions: 50 mg photocatalyst, 50 mL of pure water, O_2_ atmosphere.

**Figure S23.** Photocatalytic H_2_O_2_ production rate under different alcohol sacrificial agents. Photocatalytic conditions: 5 mg photocatalyst, 50 mL of aqueous solution, O_2_ atmosphere.

**Figure S24.** Calibration curve of benzaldehyde obtained by GC-FID analysis.

**Figure S25.** GC-FID chromatogram of the organic phase after photocatalytic reaction using mesitylene as the internal standard. The enlarged region shows the peaks assigned to benzaldehyde and mesitylene.

**Figure S26.** PXRD patterns of COF-Hzs before and after photocatalytic reaction.

**Figure S27.** FT-IR spectra of COF-Hzs before and after photocatalytic reaction.

**Figure S28.** High-resolution XPS spectra of COF-Hz-H before (upper) and after (lower) photocatalytic reaction.

**Figure S29.** High-resolution XPS spectra of COF-Hz-OMe before (upper) and after (lower) photocatalytic reaction.

**Figure S30.** High-resolution XPS spectra of COF-Hz-OEt before (upper) and after (lower) photocatalytic reaction.

**Figure S31.** High-resolution XPS spectra of COF-Hz-OEtOMe before (upper) and after (lower) photocatalytic reaction.

**Figure S32.** The amount of H_2_ during the photocatalytic system. Photocatalytic conditions: 5 mg photocatalyst, 50 mL of pure water, N_2_ atmosphere.

**Figure S33.** EPR spectra for the detection of ·O_2_^−^ using DMPO under dark and visible-light irradiation conditions.

**Figure S34.** EPR spectra for the detection of ·OH using DMPO under dark and visible-light irradiation conditions.

**Figure S35.** EPR spectra for the detection of ^1^O_2_ using TEMP under dark and visible-light irradiation conditions.

**Figure S36.** The amount of photocatalytic O_2_ evolution under a vacuum atmosphere using 0.02 M AgNO_3_ as scavengers. Photocatalytic conditions: 5 mg photocatalyst, 50 mL of water, vacuum atmosphere.

**Figure S37.** Photocatalytic H_2_O_2_ production rate by COF-Hzs under N_2_ atmosphere with different sacrificial agents (10 mM KBrO_3_, 10 % EtOH).

**Figure S38.** (a) GC-MS spectrum after photocatalysis of COF-Hz-OEtOMe with H_2_^18^O as the raw material. (b) GC-MS spectrum after decomposition with MnO_2_. It should be pointed out that the ordinary ^16^O_2_ comes from the environmental background.

**Figure S39.** The adsorption models and energies for O_2_ molecular on different sites of COF-Hz-OEtOMe.

**Figure S40.** Gibbs free energy models for intermediates *O_2_, *OOH, and *HOOH on COF-Hzs for the two-electron ORR.

**Figure S41.** Electrostatic potential surface (EPS) of COF-Hzs.

**Figure S42.** FT-IR spectra of COF-Hz-OBu.

**Figure S43.** PXRD pattern of CPOF-Hz-OBu.

**Figure S44.** Photocatalytic performance of COF-Hz-OEtOMe and COF-Hz-OBu.

**Figure S45.** The temperature of COF-Hz-OEtOMe suspension photocatalytic system under natural sunlight on a sunny day.

**Section 5. Tables S1-S****9**

**Table S1.** Materials studio simulated cell parameters of COF-Hzs.

| **COF-Hzs** | **Space group** | ***a* (Å)** | ***b* (Å)** | ***c* (Å)** | ***α* (°)** | ***β* (°)** | ***γ* (°)** |
| --- | --- | --- | --- | --- | --- | --- | --- |
| COF-Hz-H | *P3* | 37.1367 | 37.1367 | 3.5415 | 90 | 90 | 120 |
| COF-Hz-OMe | *P3* | 37.2063 | 37.2063 | 3.5256 | 90 | 90 | 120 |
| COF-Hz-OEt | *P3* | 37.2020 | 37.2020 | 3.5411 | 90 | 90 | 120 |
| COF-Hz-OEtOMe | *P3* | 37.1984 | 37.1984 | 3.5466 | 90 | 90 | 120 |

**Table S2.** Elemental analysis of COF-Hzs.

| **Samples** | **C [****wt%]** | | **N [wt%]** | | **S [wt%]** | | | **O [wt%]** | | **H [wt%]** | |
| --- | --- | --- | --- | --- | --- | --- | --- | --- | --- | --- | --- |
|  | Cal. | Exp. | Cal. | Exp. | Cal. | Exp. | Cal. | | Exp. | Cal. | Exp. |
| COF-Hz-H | 57.14 | 52.30 | 14.81 | 9.07 | 16.93 | 18.14 | 8.47 | | 18.98 | 2.65 | 2.72 |
| COF-Hz-OMe | 54.79 | 50.42 | 12.79 | 8.44 | 14.61 | 15.29 | 14.61 | | 20.99 | 3.20 | 3.08 |
| COF-Hz-OEt | 56.65 | 52.15 | 12.02 | 8.83 | 13.73 | 15.15 | 13.74 | | 19.66 | 3.86 | 3.50 |
| COF-Hz-OEtOMe | 54.75 | 53.14 | 10.65 | 8.44 | 12.17 | 14.27 | 18.25 | | 22.08 | 4.18 | 4.02 |

**Table S3.** AQY of COF-Hz-H using four band pass filters.

| **λ (nm)** | **P (mW cm^−2^)** | **C (1 h)** | **AQY** |
| --- | --- | --- | --- |
| 400 | 17.4 | 18.49 μmol | 0.90% |
| 450 | 17.9 | 18.42 μmol | 0.78% |
| 500 | 22.3 | 17.28 μmol | 0.53% |
| 550 | 24.9 | 13.14 μmol | 0.33% |
| 600 | 21.6 | 5.35 μmol | 0.14% |

**Table S4.** AQY of COF-Hz-OMe using four band pass filters.

| **λ (nm)** | **P (mW cm^−2^)** | **C (1 h)** | **AQY** |
| --- | --- | --- | --- |
| 400 | 17.4 | 43.75 μmol | 2.13% |
| 450 | 17.9 | 42.79 μmol | 1.80% |
| 500 | 22.3 | 41.13 μmol | 1.25% |
| 550 | 24.9 | 23.64 μmol | 0.59% |
| 600 | 21.6 | 9.18 μmol | 0.24% |

**Table S5.** AQY of COF-Hz-OEt using four band pass filters.

| **λ (nm)** | **P (mW cm^−2^)** | **C (1 h)** | **AQY** |
| --- | --- | --- | --- |
| 400 | 17.4 | 46.42 μmol | 2.26% |
| 450 | 17.9 | 43.38 μmol | 1.83% |
| 500 | 22.3 | 43.27 μmol | 1.32% |
| 550 | 24.9 | 27.08 μmol | 0.67% |
| 600 | 21.6 | 9.94 μmol | 0.26% |

**Table S6.** AQY of COF-Hz-OEtOMe using four band pass filters.

| **λ (nm)** | **P (mW cm^−2^)** | **C (1 h)** | **AQY** |
| --- | --- | --- | --- |
| 400 | 17.4 | 59.67 μmol | 2.91% |
| 450 | 17.9 | 60.38 μmol | 2.54% |
| 500 | 22.3 | 63.18 μmol | 1.92 % |
| 550 | 24.9 | 48.91 μmol | 1.21% |
| 600 | 21.6 | 14.92 μmol | 0.39% |

**Table S7**. The summary of the H_2_O_2_ photosynthesis performance of COF-based photocatalysts.

| Catalysts | Dosage  (mg) | Reaction solution | Light Source  (W) | Filter  (nm) | SCC | H_2_O_2_ rate  (μmol g^−1^ h^−1^) | Ref. |
| --- | --- | --- | --- | --- | --- | --- | --- |
| COF-Hz-OEtOMe | 1 | H_2_O | Xe lamp (300) | ≥ 420 | 0.62 | 7240 | This work |
|  | 5 | H_2_O | Xe lamp (300) | ≥ 420 |  | 5230 |  |
|  | 5 | H_2_O:BA  =9:1 | Xe lamp (300) | ≥ 420 |  | 22913 |  |
| TAPD-(Me)_2_ COF | 20 | H_2_O:EtOH  =1:9 | Xe lamp (300) | ≥ 420 | - | 235 | ^[13]^ |
| COF-N32 | 1 | H_2_O | Xe lamp (300) | ≥ 420 | 0.31 | 3170 | ^[14]^ |
|  | 25 | H_2_O | Sunlight | Sunlight |  | 602 |  |
| COF-2CN | 1 | H_2_O | Xe lamp (300) | ≥ 420 | 0.60 | 4858 | ^[15]^ |
| TaptBtt | 15 | H_2_O | Xe lamp (300) | ≥ 420 | 0.296 | 1407 | ^[16]^ |
| Py-Da-COF | 5 | H_2_O | Xe lamp (300) | ≥ 420 | 0.09 | 461 | ^[17]^ |
|  | 5 | H_2_O:BA  =1:1 | Xe lamp (300) | ≥ 420 |  | 3670 |  |
| DMCR-1NH | 5 | H_2_O:IPA  =10:1 | Xe lamp (300) | ≥ 420 | - | 2588 | ^[18]^ |
| TPB-DMTP-COF | 10 | H_2_O | Xe lamp (300) | ≥ 420 | 0.76 | 2882 | ^[19]^ |
| PD^2+^-COF_16.7_ | 5 | H_2_O | Xe lamp (300) | AM 1.5G | 0.34 | 1732 | ^[20]^ |
|  |  | H_2_O:EtOH  =9:1 | Xe lamp (300) | ≥ 400 |  | 11965 |  |
| CHF-DPDA | 40 | H_2_O | Xe lamp (300) | ≥ 420 | 0.78 | 1725 | ^[21]^ |
| FS-COFs | 5 | H_2_O | Xe lamp (300) | ≥ 420 | - | 3904 | ^[22]^ |
| TpDz | 3 | H_2_O | Xe lamp (300) | ≥ 420 | 0.62 | 7327 | ^[23]^ |
| TZ-COF | 45 | H_2_O | Xe lamp (300) | ≥ 420 | 0.036 | 268 | ^[24]^ |
|  | 45 | H_2_O:BA  =1:1 | Xe lamp (300) | ≥ 420 |  | 4951 |  |
| TD-COF | 1 | H_2_O | White LED | ≥ 400 | 0.15 | 4620 | ^[25]^ |
| TT-COF | 1 | H_2_O | White LED | ≥ 400 | 0.14 | 4250 |  |
| TTF-BT-COF | 5 | H_2_O | Xe lamp (300) | ≥ 420 | 0.49 | 2760 | ^[26]^ |
| TF_50_-COF | 5 | H_2_O:EtOH  =9:1 | Xe lamp (300) | ≥ 400 | 0.17 | 1739 | ^[27]^ |
| TpAQ-COF-12 | 10 | H_2_O | Xe lamp (300) | ≥ 420 | - | 420 | ^[28]^ |
| PMCR-1 | 10 | H_2_O | Xe lamp (300) | ≥ 420 | - | 1294 | ^[29]^ |
|  | 10 | H_2_O:BA  =10:1 | Xe lamp (300) | ≥ 420 |  | 5499 |  |
| 4PE-N-S | 5 | H_2_O | Xe lamp (300) | ≥ 420 | - | 1574 | ^[30]^ |
|  | 5 | H_2_O:EtOH  =9:1 | Xe lamp (300) | ≥ 420 | - | 2237 |  |
| HEP-TAPT-COF | 50 | H_2_O | Xe lamp (300) | ≥ 420 | 0.65 | 1750 | ^[31]^ |
| CTF-BDDBN | 30 | H_2_O | Xe lamp (300) | ≥ 420 | 0.14 | 97 | ^[32]^ |
| BBTz | 5 | H_2_O | Xe lamp (300) | ≥ 365 |  | 7274 | ^[33]^ |
| Hz-TP-BT-COF | 3 | H_2_O | Xe lamp (300) | 420~600 | - | 6500 | ^[34]^ |
| Hz-TP-BT-COF | 3 | H_2_O+EtOH | Xe lamp (300) | 420~600 | - | 7100 |  |
| SO_3_H-COF | 5 | H_2_O | Xe lamp (300) | ≥ 400 | 0.4 | 3015 | ^[35]^ |
|  | 5 | H_2_O:MeOH  =9:1 | Xe lamp (300) | ≥ 400 |  | 4971 |  |
| TFPA-TAPT-COF-Q | 10 | H_2_O:BA  =10:1 | Xe lamp (300) | ≥ 300 | - | 11832 | ^[36]^ |
| CN-PDA | 30 | H_2_O | Xe lamp (300) | 400~780 | 0.14 | 710 | ^[37]^ |
| FS-OHOMe-COF | 10 | H_2_O | Xe lamp (300) | ≥ 420 | 0.58 | 1480 | ^[38]^ |
|  | 10 | H_2_O:BA  =9:1 | Xe lamp (300) | ≥ 420 | - | 3000 |  |
| BTT-H3 | 5 | H_2_O | Blue LED (40) | 467 | 2.02 | 1770 | ^[12]^ |
|  | 5 | H_2_O:BA  =9:1 | Blue LED (40) | 467 | - | 2164 |  |
| DT_2_TA-TAPB | 5 | H_2_O | Xe lamp (300) | ≥ 420 | - | 8587 | ^[39]^ |
|  | 5 | H_2_O:MeOH  =9:1 | Xe lamp (300) | ≥ 420 | - | 10972 |  |
| PyNTB-2SO_3_ | 2 | H_2_O | Xe lamp (300) | ≥ 420 | - | 2764 | ^[40]^ |
|  | 2 | H_2_O:BA  =9:1 | Xe lamp (300) | ≥ 420 | - | 15158 |  |
| BT-COF | 8 | H_2_O | Xe lamp (300) | ≥ 420 | - | 4524 | ^[41]^ |
| COF-TPB | 3 | H_2_O | Xe lamp (300) | ≥ 400 | - | 1205 | ^[42]^ |
| COF-JLU90 | 5 | H_2_O | Xe lamp (300) | ≥ 300 | 1.52 | 10999 | ^[43]^ |
| ACOF-S-EtOH | 1 | H_2_O | Xe lamp (300) | AM 1.5G | - | 5440 | ^[44]^ |

**Table S8.** Life cycle inventory (LCI) data for the synthesis of COF-Hz-OEtOMe.

| **Synthesis Process** | | |
| --- | --- | --- |
| Chemicals and energy | Use | Waste |
| Benzo[1,2-b:3,4-b':5,6-b'']trithiophene-2,5,8-tricarbaldehyde | 105.6 mg, 0.32 mmol | 0 |
| 2,5-diethoxyterephthalohydrazide | 165.6 mg, 0.48 mmol | 0 |
| DMSO | 8.76 g, 8 mL | 0 |
| 6 M acetic acid | 2.4 mL (acetic acid 0.84 mL) | 0 |
| Acetone | 200 mL | 0 |
| Tetrahydrofuran | 200 mL | 0 |
| Ethanol | 200 mL | 0 |
| Energy consumption (oil bath) | 650 W (72 h) | 0 |
| Oven | 450 W (8 h) | 0 |
| **Photocatalytic Process (1 h)** | | |
| O_2_ | 600 mL (60 mL min^-1^ * 10 min) | 0 |
| **Pure H_2_O** | 50 mL, 50 g | 0 |
| COF | 5 mg | 0 |
| Product H_2_O_2_ | 0.86 mg, 25.25 μmol | 0 |
| Circulating water (Tap water) | 1 L | 0 |
| Energy consumption | 300 W | 0 |
| **Photocatalytic under natural sunlight (1 h)** | | |
| COF | 100 mg | 0 |
| **Tap H_2_O** | 1000 g, 1 L | 0 |
| H_2_O_2_ | 17.3 mg, 508 μmol | 0 |
| Energy consumption (magnetic stirrer) | 3 W | 0 |

**Table S9.** Greenhouse gas (GHG) emissions (in kg CO_2_-eq) comparison of 1 mg H_2_O_2_ production under Xe-lamp irradiation and natural sunlight at different photocatalyst synthesis scales.

| **Impact category** | **Baseline** **synthesis**  **Xe lamp** | **Fourfold-scale synthesis**  **Xe lamp** | **Baseline synthesis**  **Natural sunlight** | **Fourfold-scale synthesis**  **Natural sunlight** |
| --- | --- | --- | --- | --- |
| **Fossil** | 0.3335998 | 0.3155234 | 0.0788 | 0.060860335 |
| **Biogenic** | 0.0081381 | 0.0077747 | 0.003 | 0.002645509 |
| **Land transformation** | 0.0004077 | 0.0003835 | 7E-05 | 4.23797E-05 |
| **CO_2_ uptake** | -0.007524 | -0.007186 | -0.003 | -0.002422375 |
| **Total** | 0.334622 | 0.3164957 | 0.0791 | 0.061125849 |

References

[1]	J. P. Perdew, K. Burke, M. Ernzerhof, “Generalized Gradient Approximation Made Simple,” *Physical Review Letters* **77** (1996): 3865-3868.

[2]	S. Grimme, “Semiempirical GGA-type density functional constructed with a long-range dispersion correction,” *Journal of Computational Chemistry* **27** (2006): 1787-1799.

[3]	J. VandeVondele, J. Hutter, “Gaussian basis sets for accurate calculations on molecular systems in gas and condensed phases,” *The Journal of Chemical Physics* **127** (2007): 114105.

[4]	T. Lu, F. Chen, “Multiwfn: a multifunctional wavefunction analyzer,” *Journal of Computational Chemistry* **33** (2012): 580-592.

[5]	W. Humphrey, A. Dalke, K. Schulten, “VMD: visual molecular dynamics,” *Journal of Molecular Graphics* **14** (1996): 33-38.

[6]	A. D. Becke, “Density-functional thermochemistry. III. The role of exact exchange,” *The Journal of Chemical Physics* **98** (1993): 5648-5652.

[7]	C. Lee, W. Yang, R. G. Parr, “Development of the Colle-Salvetti correlation-energy formula into a functional of the electron density,” *Physical Review B: Condensed Matter* **37** (1988): 785-789.

[8]	F. Weigend, R. Ahlrichs, “Balanced basis sets of split valence, triple zeta valence and quadruple zeta valence quality for H to Rn: Design and assessment of accuracy,” *Physical Chemistry Chemical Physics* **7** (2005): 3297-3305.

[9]	S. Grimme, S. Ehrlich, L. Goerigk, “Effect of the damping function in dispersion corrected density functional theory,” *Journal of Computational Chemistry* **32** (2011): 1456-1465.

[10]	J. Tomasi, B. Mennucci, R. Cammi, “Quantum mechanical continuum solvation models,” *Chemical Reviews* **105** (2005): 2999-3093.

[11]	T. Lu, Q. Chen, “Shermo: A general code for calculating molecular thermochemistry properties,” *Computational and Theoretical Chemistry* **1200** (2021): 113249.

[12]	A. Chakraborty, A. Alam, U. Pal, A. Sinha, S. Das, T. Saha-Dasgupta, et al., “Enhancing photocatalytic hydrogen peroxide generation by tuning hydrazone linkage density in covalent organic frameworks,” *Nature Communications* **16** (2025): 503.

[13]	C. Krishnaraj, H. Sekhar Jena, L. Bourda, A. Laemont, P. Pachfule, J. Roeser, et al., “Strongly Reducing (Diarylamino)benzene-Based Covalent Organic Framework for Metal-Free Visible Light Photocatalytic H_2_O_2_ Generation,” *Journal of the American Chemical Society* **142** (2020): 20107-20116.

[14]	F. Liu, P. Zhou, Y. Hou, H. Tan, Y. Liang, J. Liang, et al., “Covalent organic frameworks for direct photosynthesis of hydrogen peroxide from water, air and sunlight,” *Nature Communications* **14** (2023): 4344.

[15]	Y. Hou, P. Zhou, F. Liu, Y. Lu, H. Tan, Z. Li, et al., “Efficient Photosynthesis of Hydrogen Peroxide by Cyano-Containing Covalent Organic Frameworks from Water, Air and Sunlight,” *Angewandte Chemie International Edition* **63** (2024): e202318562.

[16]	C. Qin, X. Wu, L. Tang, X. Chen, M. Li, Y. Mou, et al., “Dual donor-acceptor covalent organic frameworks for hydrogen peroxide photosynthesis,” *Nature Communications* **14** (2023): 5238.

[17]	J. Sun, H. Sekhar Jena, C. Krishnaraj, K. Singh Rawat, S. Abednatanzi, J. Chakraborty, et al., “Pyrene-Based Covalent Organic Frameworks for Photocatalytic Hydrogen Peroxide Production,” *Angewandte Chemie International Edition* **62** (2023): e202216719.

[18]	P. Das, G. Chakraborty, J. Roeser, S. Vogl, J. Rabeah, A. Thomas, “Integrating Bifunctionality and Chemical Stability in Covalent Organic Frameworks via One-Pot Multicomponent Reactions for Solar-Driven H_2_O_2_ Production,” *Journal of the American Chemical Society* **145** (2023): 2975-2984.

[19]	L. Li, L. Xu, Z. Hu, J. C. Yu, “Enhanced Mass Transfer of Oxygen through a Gas–Liquid–Solid Interface for Photocatalytic Hydrogen Peroxide Production,” *Advanced Functional Materials* **31** (2021): 2106120.

[20]	F. Hao, C. Yang, X. Lv, F. Chen, S. Wang, G. Zheng, et al., “Photo-Driven Quasi-Topological Transformation Exposing Highly Active Nitrogen Cation Sites for Enhanced Photocatalytic H_2_O_2_ Production,” *Angewandte Chemie International Edition* **62** (2023): e202315456.

[21]	H. Cheng, H. Lv, J. Cheng, L. Wang, X. Wu, H. Xu, “Rational Design of Covalent Heptazine Frameworks with Spatially Separated Redox Centers for High-Efficiency Photocatalytic Hydrogen Peroxide Production,” *Advanced Materials* **34** (2022): e2107480.

[22]	Y. Luo, B. Zhang, C. Liu, D. Xia, X. Ou, Y. Cai, et al., B. Han, “Sulfone-Modified Covalent Organic Frameworks Enabling Efficient Photocatalytic Hydrogen Peroxide Generation *via* One-Step Two-Electron O_2_ Reduction,” *Angewandte Chemie International Edition* **62** (2023): e202305355.

[23]	Q. Liao, Q. Sun, H. Xu, Y. Wang, Y. Xu, Z. Li, et al., “Regulating Relative Nitrogen Locations of Diazine Functionalized Covalent Organic Frameworks for Overall H_2_O_2_ Photosynthesis,” *Angewandte Chemie International Edition* **62** (2023): e202310556.

[24]	Y. Mou, X. Wu, C. Qin, J. Chen, Y. Zhao, L. Jiang, et al., “Linkage Microenvironment of Azoles-Related Covalent Organic Frameworks Precisely Regulates Photocatalytic Generation of Hydrogen Peroxide,” *Angewandte Chemie International Edition* **62** (2023): e202309480.

[25]	J. Y. Yue, L. P. Song, Y. F. Fan, Z. X. Pan, P. Yang, Y. Ma, et al., “Thiophene-Containing Covalent Organic Frameworks for Overall Photocatalytic H_2_O_2_ Synthesis in Water and Seawater,” *Angewandte Chemie International Edition* **62** (2023): e202309624.

[26]	J. N. Chang, Q. Li, J. W. Shi, M. Zhang, L. Zhang, S. Li, et al., “Oxidation-Reduction Molecular Junction Covalent Organic Frameworks for Full Reaction Photosynthesis of H_2_O_2_,” *Angewandte Chemie International Edition* **62** (2023): e202218868.

[27]	H. Wang, C. Yang, F. Chen, G. Zheng, Q. Han, “A Crystalline Partially Fluorinated Triazine Covalent Organic Framework for Efficient Photosynthesis of Hydrogen Peroxide,” *Angewandte Chemie International Edition* **61** (2022): e202202328.

[28]	X. C. Zhang, J. Z. Zhang, J. Miao, X. Wen, C. Chen, B. X. Zhou, et al., “Keto-enamine-based covalent organic framework with controllable anthraquinone moieties for superior H_2_O_2_ photosynthesis from O_2_ and water,” *Chemical Engineering Journal* **466** (2023): 143085.

[29]	P. Das, J. Roeser, A. Thomas, “Solar Light Driven H_2_O_2_ Production and Selective Oxidations Using a Covalent Organic Framework Photocatalyst Prepared by a Multicomponent Reaction,” *Angewandte Chemie International Edition* **62** (2023): e202304349.

[30]	M. Deng, J. Sun, A. Laemont, C. Liu, L. Wang, L. Bourda, et al., “Extending the π-conjugation system of covalent organic frameworks for more efficient photocatalytic H2O2 production,” *Green Chemistry* **25** (2023): 3069-3076.

[31]	D. Chen, W. Chen, Y. Wu, L. Wang, X. Wu, H. Xu, et al., “Covalent Organic Frameworks Containing Dual O_2_ Reduction Centers for Overall Photosynthetic Hydrogen Peroxide Production,” *Angewandte Chemie International Edition* **62** (2023): e202217479.

[32]	L. Chen, L. Wang, Y. Wan, Y. Zhang, Z. Qi, X. Wu, et al., “Acetylene and Diacetylene Functionalized Covalent Triazine Frameworks as Metal-Free Photocatalysts for Hydrogen Peroxide Production: A New Two-Electron Water Oxidation Pathway,” *Advanced Materials* **32** (2020): e1904433.

[33]	J. Cheng, S. Wan, S. Cao, “Promoting Solar-driven Hydrogen Peroxide Production over Thiazole-based Conjugated Polymers via Generating and Converting Singlet Oxygen,” *Angewandte Chemie International Edition* **62** (2023): e202310476.

[34]	R. Liu, Y. Chen, H. Yu, M. Položij, Y. Guo, T. C. Sum, et al., “Linkage-engineered donor–acceptor covalent organic frameworks for optimal photosynthesis of hydrogen peroxide from water and air,” *Nature Catalysis* **7** (2024): 195-206.

[35]	L. Li, X. Lv, Y. Xue, H. Shao, G. Zheng, Q. Han, “Custom-Design of Strong Electron/Proton Extractor on COFs for Efficient Photocatalytic H_2_O_2_ Production,” *Angewandte Chemie International Edition* **63** (2024): e202320218.

[36]	J. R. Wang, K. Song, T. X. Luan, K. Cheng, Q. Wang, Y. Wang, et al., “Robust links in photoactive covalent organic frameworks enable effective photocatalytic reactions under harsh conditions,” *Nature Communications* **15** (2024): 1267.

[37]	Y. Deng, W. Liu, R. Xu, R. Gao, N. Huang, Y. Zheng, et al., “Reduction of Superoxide Radical Intermediate by Polydopamine for Efficient Hydrogen Peroxide Photosynthesis,” *Angewandte Chemie International Edition* **63** (2024): e202319216.

[38]	C. Shu, X. Yang, L. Liu, X. Hu, R. Sun, X. Yang, et al., B. Tan, X. Wang, “Mixed-Linker Strategy for the Construction of Sulfone-Containing D-A-A Covalent Organic Frameworks for Efficient Photocatalytic Hydrogen Peroxide Production,” *Angewandte Chemie International Edition* **63** (2024): e202403926.

[39]	Y. Ju, H. Lin, G. Tan, P. Su, Z. Wang, C. Hu, et al., “Regulating the electronic structure of covalent organic frameworks via heterocyclic isomers for highly efficient photocatalytic H_2_O_2_ generation,” *Nature Communications* **16** (2025): 5658.

[40]	T. X. Luan, Q. Wei, C. Xin, S. Y. Wong, Y. Li, Y. Zheng, et al., “Remarkably Enhancing H_2_O_2_ Photogeneration by Modulating Pore-Microenvironment of a Photoactive Covalent Organic Framework,” *Advanced Materials* **38** (2026): e15517.

[41]	K. Xiong, X. Jia, Y. Kong, J. Yang, J. Guo, S. Li, et al., “Customized Covalent Organic Frameworks with in‐Plane and Intramolecular Asymmetric Polarization for Superior Hydrogen Peroxide Photosynthesis and Aerobic Oxidation of Alcohol,” *Advanced Functional Materials* **35** (2025): 2510257.

[42]	S. Yang, D. Si, L. Zou, M. Shi, Y. Huang, R. Cao, “Engineering Photo-Controlled Dynamic Conjugation Switching in Azine-Linked Covalent Organic Frameworks for Boosted Photocatalysis,” *Angewandte Chemie International Edition* **65** (2025): e18661.

[43]	Z. Zhang, Y. Hou, S. Zhu, L. Yang, Y. Wang, H. Yue, et al., “Benzotrifuran-Based Covalent Organic Frameworks for Artificial Photosynthesis of H_2_O_2_ from H_2_O, O_2_, and Sunlight,” *Angewandte Chemie International Edition* **64** (2025): e202505286.

[44]	Q. Zuo, B. Chu, X. Ye, F. Li, L. Li, Q. Xu, “Enzyme-Click Postsynthetic Modification of Covalent Organic Frameworks for Photocatalytic H_2_O_2_ Production,” *Journal of the American Chemical Society* **147** (2025): 34681-34689.
